# Stem cells, extracellular vesicles, and cell-free strategies in pulmonary arterial hypertension: a bibliometric study, 2006–2026

**DOI:** 10.3389/fmed.2026.1965787

**Published:** 2026-10-27

**Authors:** Xinyu Xue, Hui Zhang, Yunlong Cao, Zhixin Wang, Qiuyu Song, Jing Han, Dong Wang, Yuting Wu, Qian Liu

**Affiliations:** 1 Department of Cardiology, Shandong Medical and Pharmaceutical University Hospital, Binzhou, Shandong, China; 2 Department of Cardiology, Qingdao Cardiovascular Hospital, Qingdao, Shandong, China; 3 Department of Traditional Chinese Medicine, Shandong Medical and Pharmaceutical University Hospital, Binzhou, Shandong, China

**Keywords:** bibliometric analysis, cell-free therapy, exosomes, extracellular vesicles, pulmonary arterial hypertension, stem cells

## Abstract

Pulmonary arterial hypertension (PAH) remains a high-mortality vasculopathy for which no current therapy reverses established vascular remodeling. Stem cell-based and cell-free extracellular vesicle (EV) therapies are promising disease-modifying strategies, yet prior PAH bibliometric studies have not treated this stem-cell-to-cell-free arc as a dedicated, continuous research front. We analyzed 891 articles and reviews (1 January 2006 to 15 March 2026) from the Web of Science Core Collection and PubMed using CiteSpace, VOSviewer, R bibliometrix, and logistic growth modeling. Annual output grew steeply to 2012 and has since held a level of about 50 publications a year, with no trend over 2013–2025 (slope +0.44 publications per year, 95% CI −0.49 to +1.37). Cumulative output followed an S-shaped logistic trajectory (*R*^2^ = 0.860), but the fitted saturation capacity is not identified by these data and is reported as indicative only. Co-citation, clustering and keyword-burst analyses support a descriptive three-phase framework of the field's development: endothelial progenitor cell-based replacement (2006–2012), the expansion of mesenchymal stromal cell (MSC) research (2013–2021), and a cell-free EV/exosome phase (2022–2026) marked by the strongest keyword burst in the dataset (extracellular vesicles, 12.55) and by active bursts for exosomes (6.80) and endothelial dysfunction (6.57). The United States (273) and China (223) led output, with Stanford University, Stewart D.J., and Humbert M. anchoring the knowledge network. Manufacturing standardization, targeting efficiency, and large-scale clinical evidence remain unresolved, and no phase III trial has been completed for any stem cell- or EV-based product. To our knowledge, these findings provide the first quantitative mapping of this stem-cell-to-cell-free transition in PAH, and identify the directions in which research attention is concentrating.

## 1 Introduction

Pulmonary arterial hypertension (PAH) is a progressive and fatal vasculopathy characterized by obliterative remodeling of the distal pulmonary arterioles, elevated pulmonary vascular resistance (PVR), and ultimately right ventricular failure (1, 2). Despite therapeutic advances, registry data report 5-year survival rates at approximately 55%–65% (3), and the global burden of disease continues to rise (4). The etiology is multifactorial, encompassing idiopathic, heritable, drug- and toxin-induced, and connective tissue disease-associated forms, yet the final common pathway involves endothelial dysfunction, smooth muscle cell hyperproliferation, perivascular inflammation, and progressive obliteration of the pulmonary microvasculature (1, 2).

No currently approved therapy reverses established pulmonary vascular remodeling in PAH (2, 5). The FDA-approved activin signaling inhibitor sotatercept (6, 7) slows disease progression but cannot restore vascular integrity, underscoring a persistent need for disease-modifying regenerative approaches (5, 7).

In response, stem cell-based regenerative strategies have been actively explored for over 15 years. Early preclinical work with endothelial progenitor cells (EPCs) demonstrated partial repair of injured pulmonary endothelium (8), followed by mesenchymal stromal cell (MSC) transplantation whose therapeutic benefit was increasingly attributed to paracrine signaling rather than direct engraftment—a mechanistic pivot that fundamentally reshaped the field (9–11). The recognition that MSC-derived EVs recapitulate the anti-inflammatory, anti-proliferative, and pro-angiogenic effects of parent cells, while potentially circumventing risks of uncontrolled differentiation and pulmonary sequestration, has accompanied a rapid transition toward cell-free strategies over the past 5 years (12, 13). This paradigm migration—from cell-based EPC therapy to cellular secretion products (MSC-conditioned medium) to nanoparticle-scale EV therapeutics—represents a coherent evolutionary trajectory spanning two decades that has not, to our knowledge, been systematically mapped from a bibliometric standpoint.

The mechanistic basis of MSC therapy in PAH extends across multiple complementary modalities. MSCs secrete growth factors that support endothelial survival and angiogenesis while exerting broad immunomodulatory effects (14). MSC-derived EVs carry bioactive microRNA and protein cargos that recapitulate these effects, partly by polarizing pulmonary macrophages toward an anti-inflammatory M2 phenotype (15). Beyond secreted factors, MSCs can transfer functional mitochondria to stressed pulmonary endothelial cells through tunneling nanotubes, restoring bioenergetic capacity and attenuating apoptosis (16, 17). These integrated mechanisms—growth factor secretion, EV-mediated signaling, immunomodulation, and mitochondrial donation—establish MSCs as multimodal therapeutic agents whose mechanistic complexity is increasingly reflected in the evolving publication landscape.

Prior bibliometric analyses of the PAH literature have each addressed complementary but non-overlapping questions. He et al. (18) mapped the general PAH research landscape (2011–2020) and identified “mesenchymal stem cells” as an emerging keyword, but their study covered the full breadth of PAH research without isolating stem cell/cell-free therapy as a dedicated analytical target. Other adjacent efforts focused on hypoxic pulmonary hypertension (19), chronic thromboembolic pulmonary hypertension (20), and artificial intelligence applications in PH (21)—none interrogated the stem cell or cell-free therapy literature as a primary object. A dedicated bibliometric study that treats the full arc of stem cell-to-cell-free evolution in PAH—spanning two decades, capturing the recent EV explosion, and quantifying the paradigm transition through formal growth modeling—remains, to our knowledge, absent.

To address this gap, the present study constructs and analyzes a curated corpus of 891 publications on stem cell and cell-free therapeutic strategies for PAH published between 2006 and 2026. Bibliometric analyses were conducted using CiteSpace, VOSviewer, R bibliometrix, and SCImago Graphica, augmented by logistic growth modeling. This study was guided by five research questions targeting: RQ1, temporal publication and citation trends and the quantifiability of the cell-to-cell-free paradigm shift; RQ2, core knowledge-producing entities and their collaboration patterns; RQ3, the intellectual structure and milestone publications driving paradigm shifts; RQ4, thematic evolution across the EPC, MSC and EV/exosome phases; and RQ5, emerging research frontiers and their translational implications. Through systematic multi-dimensional cross-analysis, this study aims to reveal the internal logic of the research paradigm evolution from cell replacement through paracrine mechanisms to engineered cell-free therapy, and to formulate evidence-grounded assessments of future trajectories that may inform research direction selection and clinical trial design in cell and gene therapy for PAH.

## 2 Materials and methods

### 2.1 Data sources and search strategy

Data were retrieved from the Web of Science Core Collection (WoSCC) and PubMed databases. A comprehensive systematic literature search was conducted on 15 March 2026 to identify publications related to stem cells, extracellular vesicles, and cell-free therapies for pulmonary arterial hypertension (PAH). All records available up to 15 March 2026 were retrieved. Only articles and reviews published in English were included. (Figure 1, Table 1).

**Figure 1:**
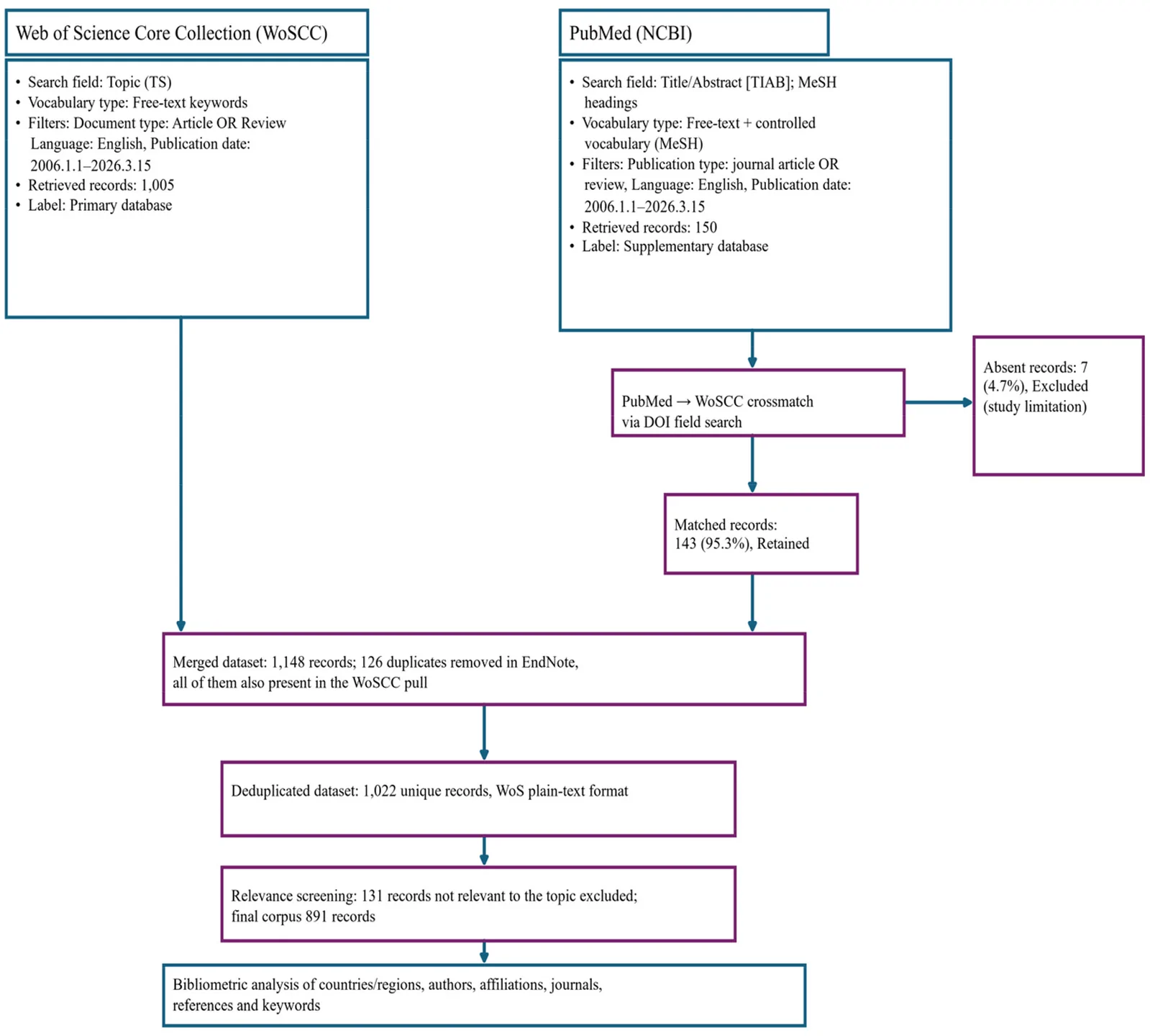
Schematic representation of data collection and processing, 2006.1.1–2026.3.15.

**Table 1:** Search strategy and retrieval results (search date: 15 march 2026).

Database	Search field	Vocabulary type	Filters applied	Records (*n*)	Notes
Web of Science Core Collection (WoSCC)	Topic (TS)	Free-text keywords	Document type: Article OR Review; language: English; publication date: 2006.1.1–2026.3.15	1,005	Primary database. 1,140 identified before document-type (−127) and language (−8) filters
PubMed (NCBI)	Title/Abstract [TIAB]; MeSH headings	Free-text + controlled vocabulary (MeSH)	Publication type: journal article OR review; language: English; publication date: 2006.1.1–2026.3.15	150	Supplementary database. 701 identified before publication-type and language filters (−551)
PubMed → WoSCC cross-match	DOI field search	–	Each PubMed record verified via DOI in WoSCC; matched records exported in WoS plain-text format to ensure cited-references (CR) field integrity	143 (95.3%)	Matched and retained
PubMed records absent from WoSCC	–	–	Not retrievable in WoSCC; excluded to maintain format consistency	7 (4.7%)	Excluded (study limitation)
Merged dataset	–	–	WoSCC (1,005) + PubMed-matched (143); duplicates removed in EndNote	1,148 (pre-dedup)	–
Deduplicated dataset	–	–	Unique records in WoS plain-text format	1,022	126 duplicate records removed
Relevance screening (PAH scope)	–	–	Records carrying no PAH or PH term in the title, author keywords or Keywords Plus were read in full; those addressing conditions outside the pulmonary circulation were excluded	1,022 → 891	131 records excluded (12.8%)
Final dataset for bibliometric analysis	–	–	Records in WoS plain-text format, scoped to pulmonary arterial hypertension and pulmonary hypertension	891	Used for all analyses in this study

The search strategy for both databases was structured around two concept groups connected by the Boolean operator AND: Concept Group A encompassing stem cells, extracellular vesicles, and cell-free therapies (including mesenchymal stem cells, endothelial progenitor cells, induced pluripotent stem cells, bone marrow-derived cells, adipose- and umbilical cord-derived stem cells, extracellular vesicles, exosomes, conditioned medium, secretome, and gene-modified stem cells); and Concept Group B encompassing PAH and its clinical subtypes, hemodynamic parameters, relevant cell types, molecular targets, and animal models. Within each concept group, synonyms and related terms were connected by OR, with asterisks (*) used as truncation wildcards to capture morphological variants. For WoSCC, a Topic (TS) field search was performed using free-text keywords exclusively. For PubMed, an Advanced Search was conducted combining controlled vocabulary terms (Medical Subject Headings [MeSH]) with free-text keywords restricted to the title and abstract fields [TIAB] to maximise retrieval sensitivity. Both searches were limited to original research articles and reviews published in English. The 2006 lower limit bounds publications, not the references they cite: earlier foundations of the field enter the analysis through the cited-reference field of the co-citation network. The complete search strings for both databases are provided in Supplementary Table S1.

To preserve the integrity of the cited-reference field required for co-citation analysis, records were integrated with WoSCC priority. The WoSCC search returned 1,005 records and the PubMed search 150; 143 PubMed records (95.3%) were matched to WoSCC by DOI and retrieved in WoS format, and the 7 that could not be matched (4.7%) were excluded, as noted in the limitations. The pool of 1,148 records was deduplicated in EndNote to 1,022. Two authors independently screened these records for relevance to the topic and excluded those judged unrelated to PAH (*n* = 131), leaving 891 records for all analyses. The search was not restricted by clinical group: records indexed to Group 2–5 pulmonary hypertension and animal models of the disease are in scope, so the corpus is PAH-centred rather than PAH-restricted.

### 2.2 Data processing and analysis tools

VOSviewer (version 1.6.20) was used for the keyword co-occurrence, co-authorship, institutional and source networks; Scimago Graphica for the geographic map and the circular author diagram; Charticulator for the country chord diagram, whose data were exported from VOSviewer and converted with Gephi; biblioshiny for the thematic map, trend topics and author production chart; and CiteSpace (version 6.4.R2) for co-citation, cluster and burst analysis. The settings for each analysis, with the network sizes and the cluster-quality metrics modularity (Q) and silhouette (S), are reported in Supplementary Table S2. Terms were harmonized with a single thesaurus of 1,312 rules before the keyword analysis, and equivalent files were exported for the three packages. Growth was modelled with the lifeCycle function of bibliometrix (22, 23) on the complete calendar years 2006–2025, giving *R*^2^ = 0.860, an inflection point at 2020.1 and a fitted capacity of 1,391 publications (95% bootstrap CI 1,192–1,687) that varies by 129% across truncation points (607–1,391) and is therefore reported as indicative. Each record was also classified by therapeutic class from its own keywords, with compounds counted under both classes so that the shares do not sum to 100, and change over time was located with segmented regression (segmented 2.2.1 in R), the number of breakpoints chosen by BIC and checked by penalising them twofold and threefold and by the Davies test. The search strings are reproduced as executed, so Supplementary Table S1 retains the phrase “mesenchymal stem cells”; the standardized term “mesenchymal stromal cells” is used throughout.

## 3 Results

### 3.1 Overview of publications

A total of 891 publications (original articles and reviews) published between 1 January 2006 and 15 March 2026 were retrieved from the Web of Science Core Collection (WoSCC) and PubMed databases. These publications involved 5,169 authors from 976 institutions across 56 countries/regions.

Annual publication output exhibited three phases (Figure 2A). During the initial phase (2006–2012), annual output increased from 8 to 35 publications, a rise of +4.50 publications per year (95% CI 2.84–6.16). From 2013 annual output held between 45 and 65 publications, with no detectable trend over 2013–2021 (+1.08 publications per year, 95% CI −0.73 to +2.90). From 2022 onward it held between 47 and 58 publications per year. The highest single year is 2021, at 65 publications. The 13 publications recorded for 2026 reflect incomplete database indexing and do not represent an actual decline in research activity. Life cycle model fitting of annual publication output gave *R*^2^ = 0.860 (Figure 2B), with cumulative output following an S-shaped curve characteristic of scientometric dynamics. The theoretical inflection point was estimated at 2020.1, and the fitted saturation capacity was 1,391 publications (95% bootstrap CI 1,192–1,687); it varied by 129% across truncation points, so it is reported as indicative (Figure 2C). At the time of analysis, cumulative output had reached 891 records. In terms of citation impact, the 62 records published in 2017 had accumulated 5,681 citations between them as of the retrieval date, the highest annual total in the series, despite a publication count below the 2021 maximum; because publication years differ in citation window, this measure favours earlier cohorts. This non-linear decoupling between publication volume and citation accumulation suggests the emergence of high-impact foundational studies during this period. Whether the phase boundaries are supported by the data, and how the therapeutic composition of the corpus changed, are examined in Supplementary Note S1.

**Figure 2:**
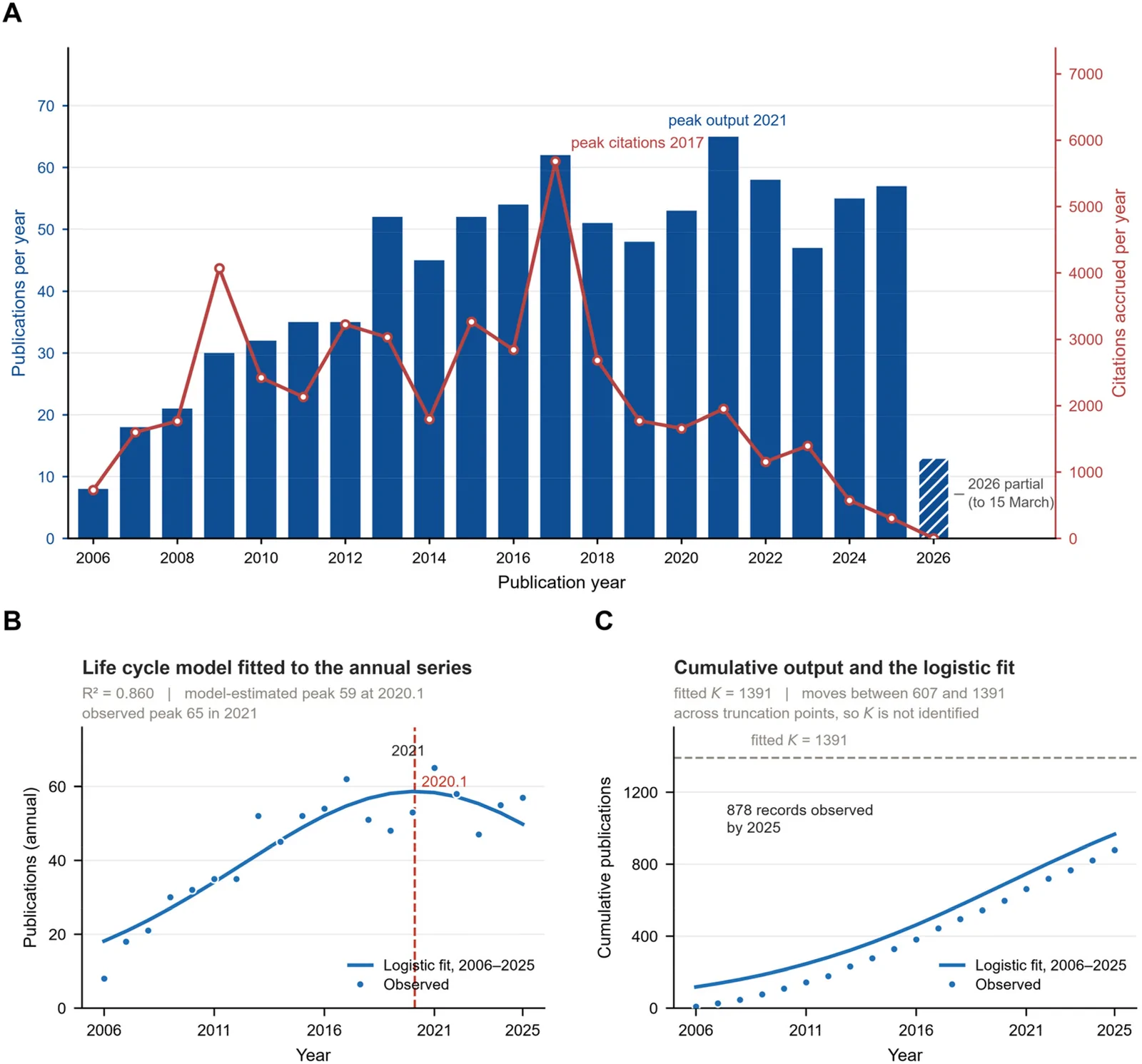
Publication trends and growth dynamics. **(A)** Annual publication volumes and annual citation counts from 2006 to 2026. **(B)** Life cycle model fitted to the annual series (*R*^2^ = 0.860). The model-estimated annual peak is 59 publications at 2020.1, to be distinguished from the observed peak of 65 publications in 2021. **(C)** Cumulative output with the same fit (saturation *K* = 1,391; 95% bootstrap CI 1,192–1,687). Neither panel is extrapolated beyond 2025: because the fitted capacity varies by 129% across truncation points (607–1,391), it is read as indicative and no forecast is drawn from it.

### 3.2 Contributions of countries and regions

Fifty-six countries/regions contributed to the literature in this field. The United States (273 publications, 30.64%) and China (223 publications, 25.03%) were the two largest contributors, together accounting for 55.67% of total output (Table 2). The United States led in total citations (17,835) and total link strength (TLS = 235) in the collaboration network, occupying a central hub position (Figures 3A,C).

**Table 2:** Top 10 countries by corresponding-author publications.

Rank	Country	Articles	Articles %	SCP	MCP	MCP %	TC	Average article citations	Total link strength
1	USA	273	30.64	208	65	23.81	17,835	65.3	235
2	China	223	25.03	190	33	14.80	5,150	23.1	59
3	United Kingdom	46	5.16	26	20	43.48	4,759	103.5	131
4	France	44	4.94	21	23	52.27	2,314	52.6	111
5	Canada	41	4.60	28	13	31.71	2,621	63.9	78
6	Italy	37	4.15	27	10	27.03	990	26.8	78
7	Japan	34	3.82	30	4	11.76	1,216	35.8	32
8	Germany	31	3.48	14	17	54.84	2,123	68.5	114
9	Netherlands	23	2.58	11	12	52.17	1,317	57.3	76
10	Brazil	18	2.02	15	3	16.67	509	28.3	15

**Figure 3:**
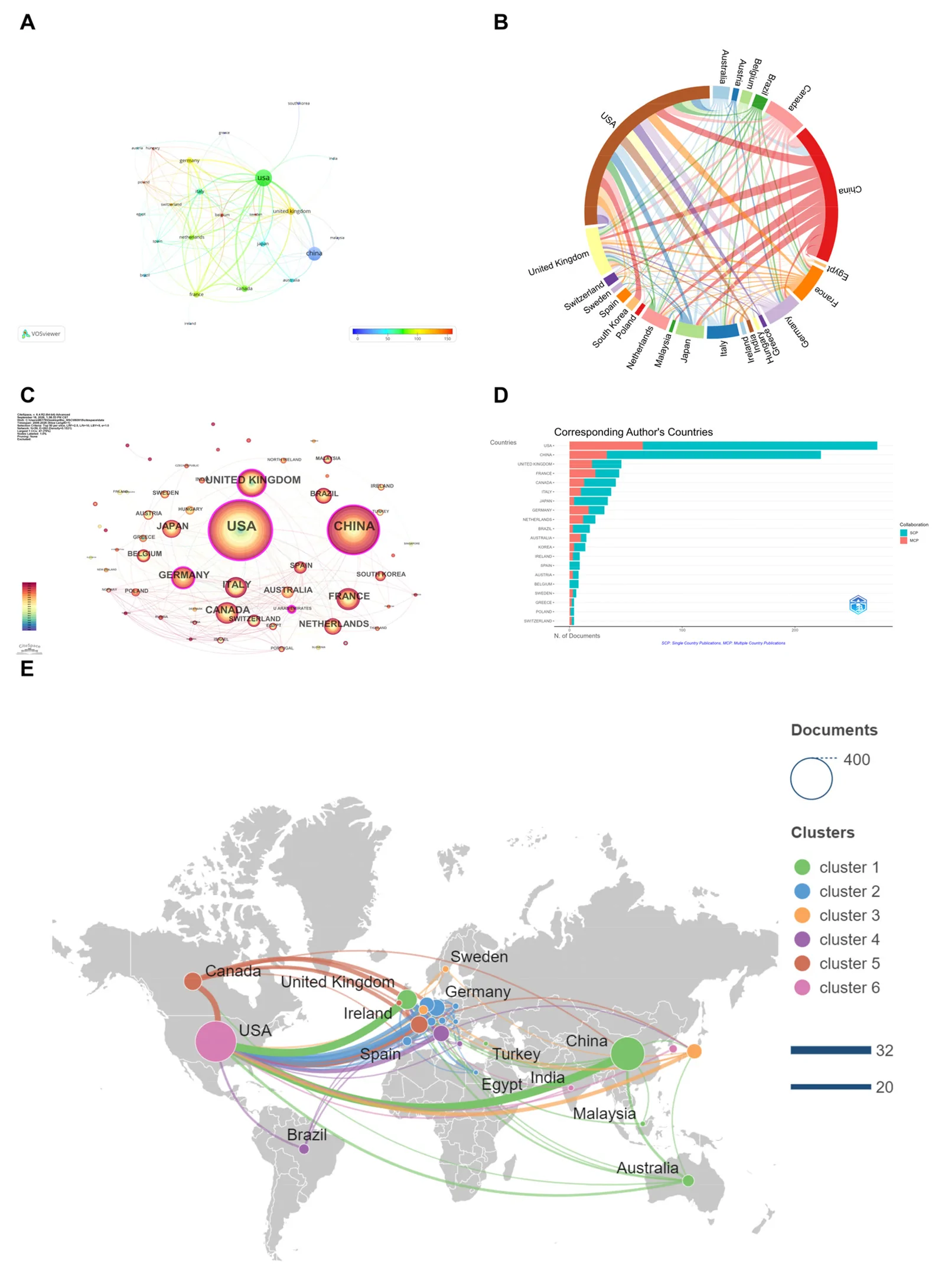
Country-level research output and international collaboration. **(A)** Co-authorship network of countries constructed via VOSviewer. Node size indicates publication volume, node color represents citation frequency, and edge thickness denotes collaboration intensity. **(B)** Chord diagram illustrating bilateral co-authorship relationships among the top contributing countries. **(C)** Country cluster map from CiteSpace showing regional research groupings. **(D)** Bar chart comparing publication volumes of the top 20 countries, with single-country (SCP) and multi-country (MCP) contributions distinguished. **(E)** Geographic map of global publication distribution and inter-country collaboration links.

Analysis of collaboration patterns revealed marked heterogeneity across countries (Figure 3D). China's multiple-country publication (MCP) ratio was 14.80%, indicating predominantly domestic collaboration. Among countries with at least ten multi-country publications, Australia (66.67%), Germany (54.84%), France (52.27%) and the Netherlands (52.17%) exceeded 50% MCP ratios. The United Kingdom recorded the highest average citation frequency (103.5 citations per publication) among major contributing countries, despite lower total output.

The international collaboration network (Figures 3B,E) showed a dense North America-Western Europe axis, with the strongest bilateral links observed between USA-China, USA-UK, and USA-Canada.

### 3.3 Contributions of authors

The author collaboration network (Figure 4A) exhibited localized high-density clustering within a relatively dispersed overall structure. Core collaborative groups were identified around Stewart Duncan J., Rabinovitch Marlene, Morrell Nicholas W., and Humbert Marc. The circular layout in Figure 4B arranges the same authors by cluster, with node size proportional to publication output.

**Figure 4:**
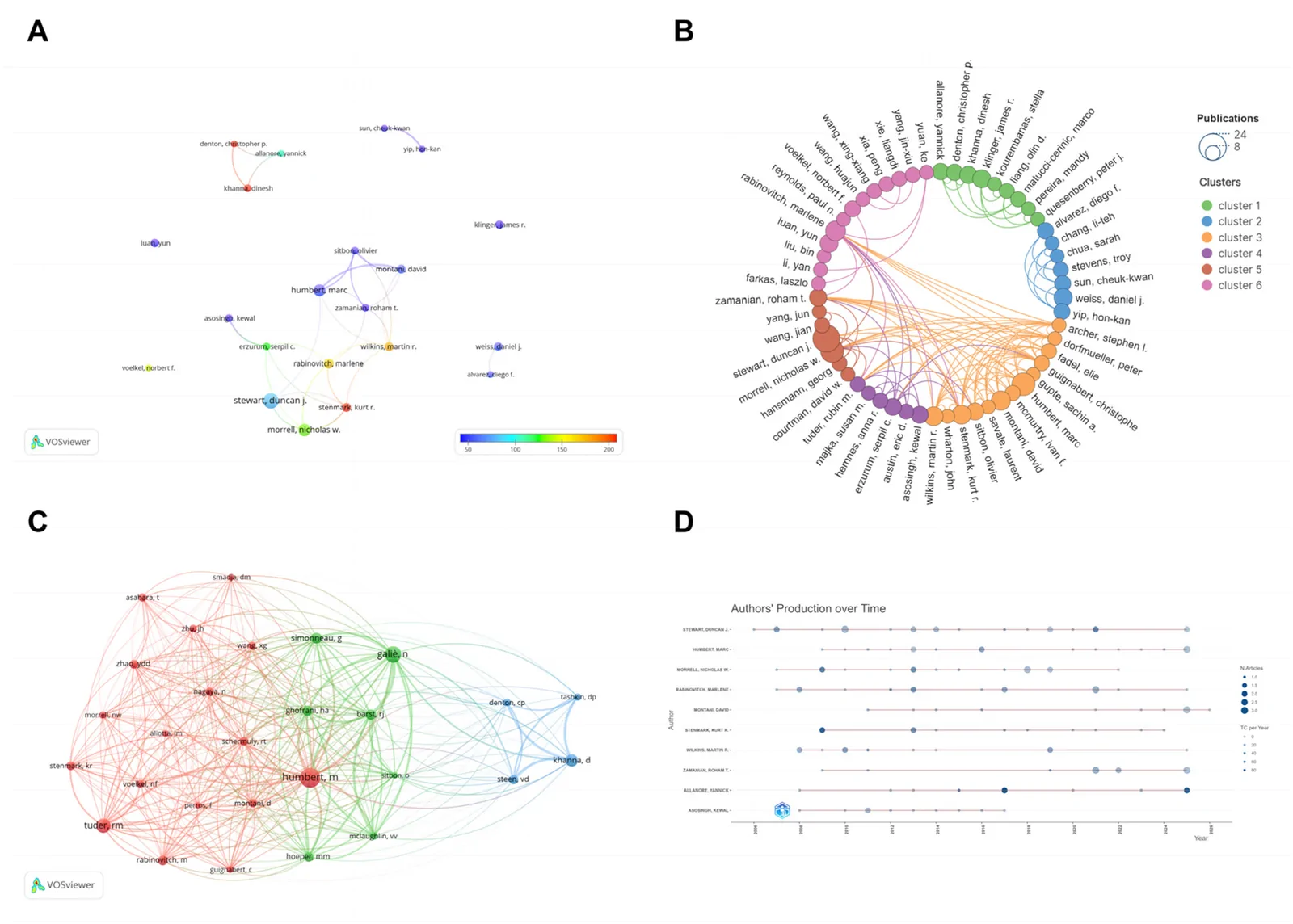
Author productivity, influence, and collaboration networks in the field. **(A)** Author co-authorship network by VOSviewer. Node size denotes publication count, color indicates citation frequency, and edges show co-authorship links. **(B)** Circular co-authorship network of core authors in the field. Authors are arranged along the circumference, with node size proportional to publication count (range: 8–24) and arc thickness reflecting collaboration frequency. Six color-coded clusters indicate distinct research communities. **(C)** Co-cited author network in the field. Node size reflects citation frequency, and link thickness indicates co-citation strength. Three clusters are identified: the red cluster (center) forms the dominant hub encompassing the most frequently co-cited authors, the green cluster (upper left) represents a secondary research community, and the blue cluster (right) constitutes a relatively independent citation group with linear connectivity. **(D)** Authors’ production over time. Each bubble represents the number of articles published by a given author in a specific year, with bubble size proportional to annual output and color intensity indicating citations per article. The timeline spans 2006–2026, covering the ten highest-output authors.

In terms of publication output and impact, Stewart Duncan J. ranked first in both total publications (22) and H-index (16), with output spanning 2006–2025 (Figure 4D, Table 3). Denton Christopher P. recorded the highest average citation frequency (360.9 citations per publication). Rabinovitch Marlene and Humbert Marc showed sustained publication activity spanning over 15 years. In recent years, Montani David showed increasing publication output, representing an emerging contributor group.

**Table 3:** Top 10 high index authors and co-cited authors.

Rank	Author	H_index	TC	Articles	Average citation per article	Rank	Co-cited author	Citations	Total link strength
1	Stewart Duncan J.	16	1,809	22	82.2	1	Humbert, M.	423	16,444
2	Rabinovitch Marlene	14	2,041	15	136.1	2	Galiè, N.	318	12,803
3	Morrell Nicholas W.	13	2,112	16	132.0	3	Tuder, R. M.	268	10,148
4	Stenmark Kurt R.	10	2,245	11	204.1	4	Khanna, D.	222	8,510
5	Wilkins Martin R.	10	1,898	11	172.5	5	Simonneau, G.	196	8,514
6	Humbert Marc	10	985	17	57.9	6	Barst, R. J.	188	10,151
7	Denton Christopher P.	9	3,609	10	360.9	7	Ghofrani, H. A.	180	9,863
8	Allanore Yannick	9	1,832	10	183.2	8	Rabinovitch, M.	171	5,775
9	Weiss Daniel J.	9	567	10	56.7	9	Hoeper, M. M.	163	6,661
10	Khanna Dinesh	8	2,856	10	285.6	10	Steen, V. D.	158	6,343

Author co-citation analysis identified Humbert M. as the most co-cited author (423 co-citations, TLS = 16,444), followed by Galiè N. and Tuder R.M. (Table 3). The co-citation network (Figure 4C) partitioned into three interacting clusters: (i) Humbert M. and Galiè N., centered on clinical diagnostic and therapeutic standards; (ii) Tuder R.M., Rabinovitch M., and Stenmark K.R., focused on basic pathophysiological and molecular mechanisms; and (iii) Khanna D. and Denton C.P., reflecting extension toward specific comorbidities. Dense cross-cluster connectivity was observed, particularly between the clinical and basic research clusters.

### 3.4 Institutional contributions

Institutional network analysis revealed a hub-and-spoke structure with Stanford University occupying the central position in both the co-authorship network (Figure 5A) and the citation network (Figure 5B). Stanford University led in publication count (40) and citations (3,259), and held the highest total link strength in the co-authorship network (63) and in the citation network (584) (Table 4). Zhejiang University ranked second in publications (32), representing the most prolific Chinese institution. The University of Colorado ranked third (29 publications), with 2,603 citations and a citation-network TLS of 474.

**Figure 5:**
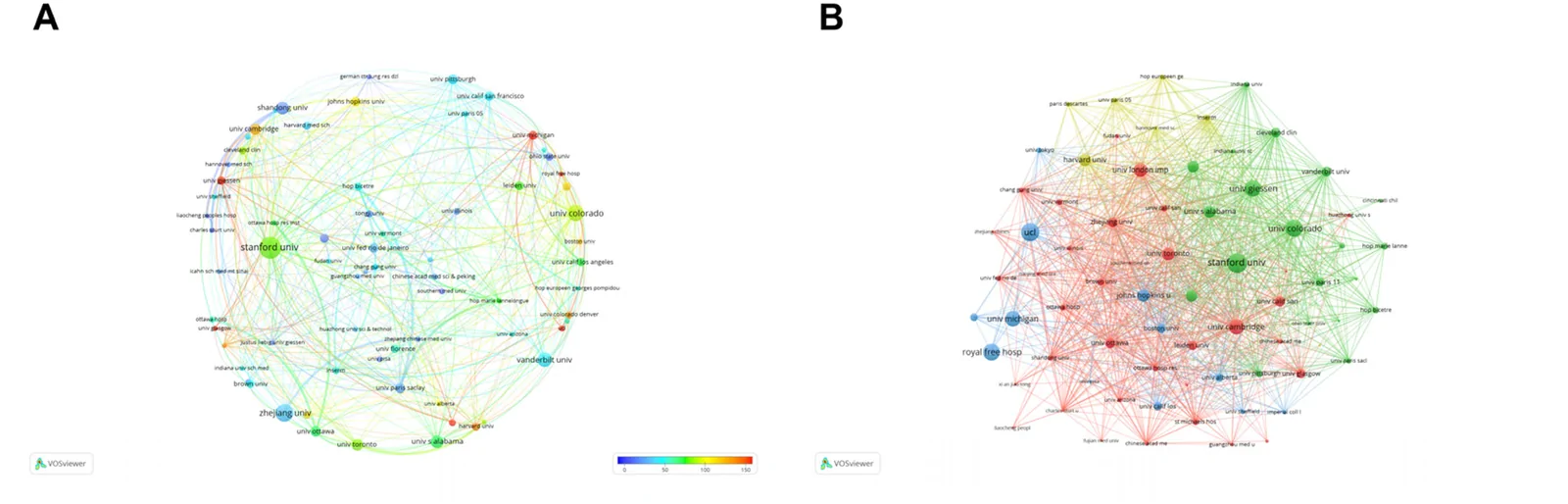
Institutional collaboration network of research contributions. **(A)** Institutional co-authorship network in the field. Node size reflects publication output, and link thickness indicates collaborative intensity. **(B)** Institutional citation network in the field, built from citation relations between organizations. Node size is proportional to citation frequency and link thickness reflects citation link strength.

**Table 4:** Top 10 organizations in the co-authorship and citation networks.

Rank	Organization (co-authorship)	Documents	Citations	Rank	Organization (citation)	Documents	Citations	Total link strength
1	Stanford Univ	40	3,259	1	Stanford Univ	40	3,259	584
2	Zhejiang Univ	32	1,095	2	UCL	9	2,895	126
3	Univ Colorado	29	2,603	3	Univ Colorado	29	2,603	474
4	Vanderbilt Univ	25	1,089	4	Royal Free Hosp	7	2,568	49
5	Univ S Alabama	22	1,526	5	Univ Giessen	13	2,432	318
6	Shandong Univ	22	461	6	Univ Cambridge	18	2,181	396
7	Univ Toronto	20	1,681	7	Univ Michigan	14	2,172	80
8	Univ Cambridge	18	2,181	8	Univ London Imperial Coll Sci Technol & Med	11	1,753	263
9	Univ Ottawa	17	1,157	9	Univ Toronto	20	1,681	300
10	Univ Pittsburgh	16	663	10	Harvard Univ	12	1,619	269

Among high-impact institutions, the University of Giessen achieved 2,432 citations from 13 publications. The highest citation-to-publication ratios among the ten most cited institutions belonged to Royal Free Hospital (2,568 citations from 7 publications) and University College London (2,895 from 9). The University of Cambridge (2,181 citations), University of Michigan (2,172 citations), and Imperial College London (1,753 citations) also showed substantial citation impact. Shandong University ranked sixth by publication count but recorded a comparatively low citation count (461). Additional high-output US institutions included Vanderbilt University, the University of South Alabama, and the University of Pittsburgh.

### 3.5 Journal contributions

The top three source journals by publication count were Pulmonary Circulation (32 publications), Circulation (22 publications), and American Journal of Physiology-Lung Cellular and Molecular Physiology (21 publications) (Figure 6A, Table 5). Circulation ranked highest in academic impact metrics: H-index (20), total citations (2,784), and 2024 impact factor (38.6, JCR Q1). American Journal of Physiology-Lung Cellular and Molecular Physiology (H-index 17, 1,612 citations) and European Respiratory Journal (H-index 16, IF = 21.0) followed. Core journals formed a dense interactive network spanning cardiovascular, respiratory, and cellular/molecular biology disciplines.

**Figure 6:**
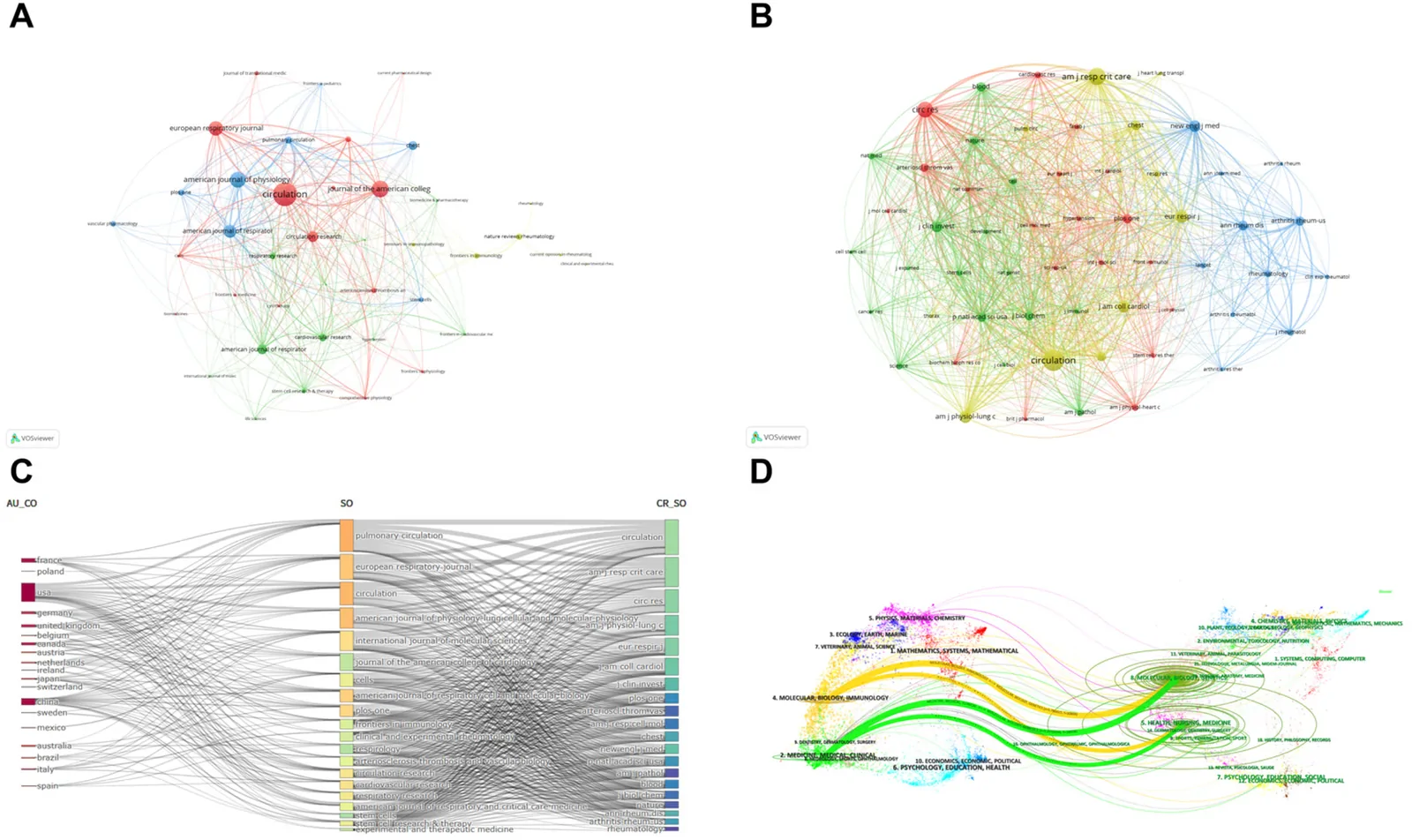
Journal contribution landscape and knowledge dissemination patterns in stem cell therapy for PAH research. **(A)** Source journal network, that is, the journals in which the corpus was published. Node size reflects publication frequency. **(B)** Co-cited journal network. Three clusters are identified: cardiovascular (red), respiratory and critical care (green), and basic science (blue), reflecting the multidisciplinary knowledge base of this field. **(C)** Sankey diagram of knowledge flow from contributing countries (AU_CO) through publishing journals (SO) to co-cited journals (CR_SO). Despite national publishing differences, co-cited journals converge on Pulmonary Circulation, Circulation, and European Respiratory Journal as shared knowledge sources. **(D)** Journal dual-map overlay. Citing journals (left) cluster in “Molecular, Biology, Immunology” and in “Medicine, Medical, Clinical”; cited journals (right) cluster in “Molecular, Biology, Genetics”. Both dominant citation paths terminate in Molecular, Biology, Genetics, one from molecular biology and immunology and one from medicine and clinical medicine, so the field, including its clinical literature, draws substantially on molecular and genetic knowledge.

**Table 5:** Top 15 journals by h-index.

Rank	H_index	TC	Journal	PY_start	Articles	Total link strength	JCR (2024)	IF (2024)
1	20	2,784	CIRCULATION	2007	22	167	Q1	38.6
2	17	1,612	AMERICAN JOURNAL OF PHYSIOLOGY-LUNG CELLULAR AND MOLECULAR PHYSIOLOGY	2008	21	109	Q1	3.5
3	16	1,418	EUROPEAN RESPIRATORY JOURNAL	2008	19	111	Q1	21.0
4	16	540	PULMONARY CIRCULATION	2,013	32	136	Q2	2.5
5	15	881	AMERICAN JOURNAL OF RESPIRATORY CELL AND MOLECULAR BIOLOGY	2006	18	93	Q1	5.3
6	13	464	PLOS ONE	2011	18	56	Q2	2.6
7	11	408	INTERNATIONAL JOURNAL OF MOLECULAR SCIENCES	2018	16	84	Q1	4.9
8	10	954	CIRCULATION RESEARCH	2006	11	79	Q1	16.2
9	10	611	CARDIOVASCULAR RESEARCH	2007	11	71	Q1	8.7
10	9	1,231	AMERICAN JOURNAL OF RESPIRATORY AND CRITICAL CARE MEDICINE	2009	9	137	Q1	19.4
11	8	380	ARTERIOSCLEROSIS THROMBOSIS AND VASCULAR BIOLOGY	2017	8	46	Q1	7.8
12	8	303	STEM CELL RESEARCH & THERAPY	2016	11	50	Q1	7.3
13	7	415	FRONTIERS IN IMMUNOLOGY	2015	8	9	Q1	5.9
14	7	144	CELLS	2020	10	63	Q2	6.0
15	6	1,759	JOURNAL OF THE AMERICAN COLLEGE OF CARDIOLOGY	2007	7	93	Q1	22.3

Co-citation journal analysis identified Circulation (3,387 co-citations, TLS = 239,248), American Journal of Respiratory and Critical Care Medicine (2,514 co-citations), and Circulation Research (2,251 co-citations) as the three most co-cited journals (Figure 6B, Tables 5, 6). Although Pulmonary Circulation led by publication volume, Circulation ranked first among source journals in academic impact metrics (H-index, total citations, impact factor) and was simultaneously the most frequently co-cited journal in the field. In the source block, total link strength is reported only for journals the source network retains, that is those with at least five documents in the corpus; the four with fewer are marked n/a.

**Table 6:** Top 10 source journals and co-cited journals.

Rank	Source journal	Documents	Citations	Total link strength	Rank	Co-cited journal	Citations	Total link strength
1	CIRCULATION	22	2,784	167	1	Circulation	3,387	239,248
2	LANCET	4	2,498	n/a	2	Am J Resp Crit Care	2,514	183,012
3	JOURNAL OF THE AMERICAN COLLEGE OF CARDIOLOGY	7	1,759	93	3	Circ Res	2,251	169,988
4	AMERICAN JOURNAL OF PHYSIOLOGY-LUNG CELLULAR AND MOLECULAR PHYSIOLOGY	21	1,612	109	4	Eur Respir J	1,582	110,143
5	EUROPEAN RESPIRATORY JOURNAL	19	1,418	111	5	Am J Physiol-Lung C	1,513	106,872
6	PHYSIOLOGICAL REVIEWS	3	1,418	n/a	6	J Am Coll Cardiol	1,432	89,195
7	AMERICAN JOURNAL OF RESPIRATORY AND CRITICAL CARE MEDICINE	9	1,231	137	7	New Engl J Med	1,392	110,694
8	ANNALS OF THE RHEUMATIC DISEASES	3	1,034	n/a	8	J Clin Invest	1,316	112,507
9	CIRCULATION RESEARCH	11	954	79	9	Ann Rheum Dis	1,123	76,850
10	NATURE REVIEWS CARDIOLOGY	3	924	n/a	10	Chest	1,045	76,473

The publishing country-source journal-co-cited journal Sankey diagram (Figure 6C) showed that the United States and China contributed the largest volume of publications to Pulmonary Circulation, Circulation, and European Respiratory Journal, while these publications' knowledge base drew most heavily from Circulation and American Journal of Respiratory and Critical Care Medicine.

The dual-map overlay (Figure 6D) places the field's citing journals in “Molecular, Biology, Immunology” and in “Medicine, Medical, Clinical”, while the cited journals concentrate in “Molecular, Biology, Genetics”. Both dominant citation paths terminate in Molecular, Biology, Genetics, one from molecular biology and immunology and one from medicine and clinical medicine. The clinical as well as the molecular citing literature therefore draws substantially on molecular and genetic knowledge, which is not a unidirectional movement from mechanistic research toward clinical application.

### 3.6 Reference co-citation analysis

The reference co-citation network (Figure 7A, Table 7) identified Zhao YD (2005) and Wang XX (2007) as the most co-cited references (151 and 122 co-citations, respectively), both occupying central positions in the network. Asahara T (1997) provided the foundational angiogenesis framework for the field. Lane KB (2000), addressing the BMPR2 gene, demonstrated strong co-citation associations with stem cell research publications.

**Figure 7:**
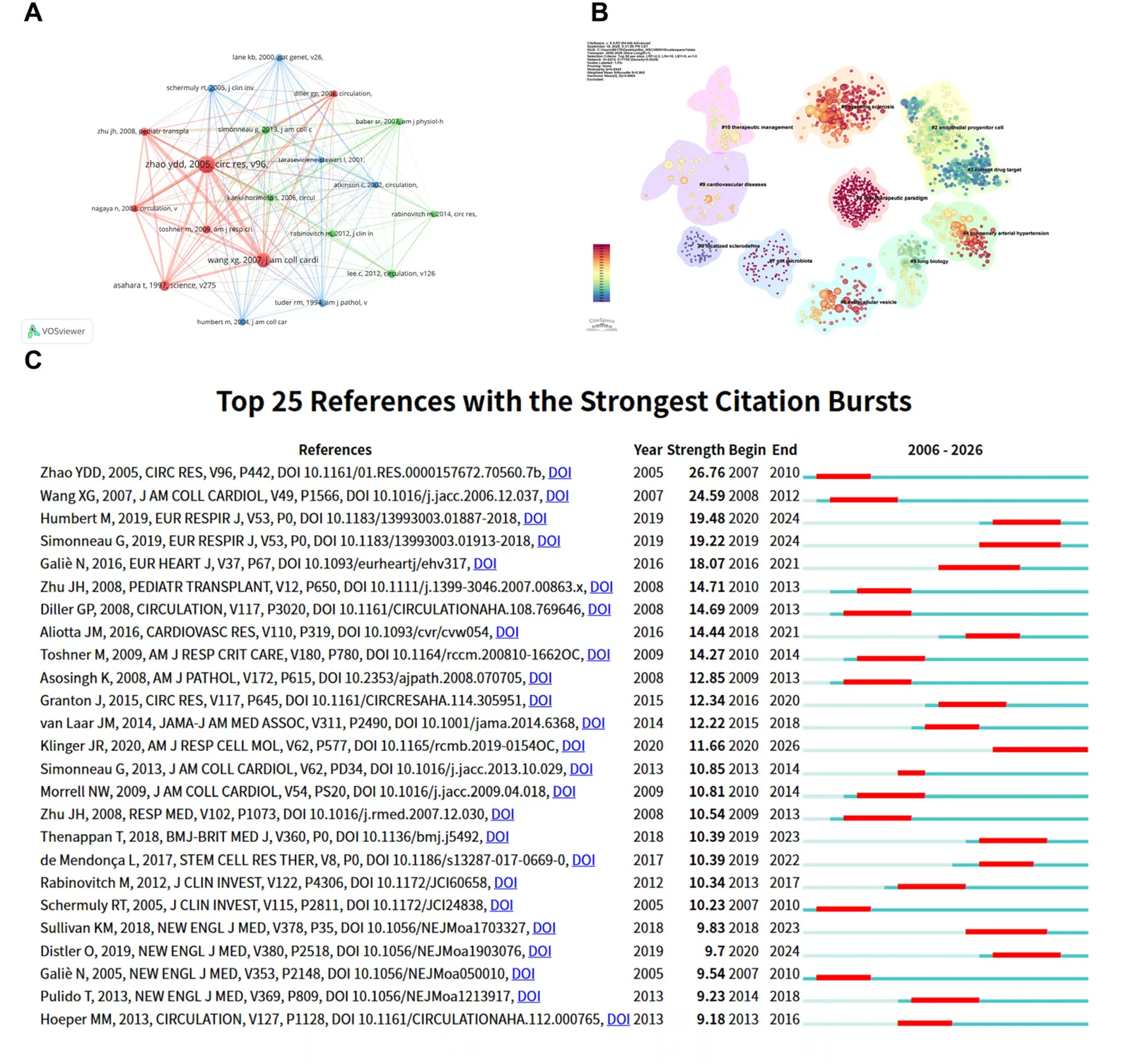
Co-cited reference analysis and research frontier evolution in stem cell therapy for PAH. **(A)** Co-cited reference network. Node size reflects citation frequency, with three clusters representing core cell therapy studies (red), clinical guidelines (blue), and emerging mechanistic research (green). **(B)** Reference cluster map (CiteSpace). Nodes are colored by publication year (blue: earlier; red: recent). Eleven clusters are identified. **(C)** Top 25 references with the strongest citation bursts (2006–2026). Cluster and burst labels are reproduced as generated by CiteSpace. The reference indexed there as Wang XG (2007, Journal of the American College of Cardiology) is the trial of autologous endothelial progenitor cell transplantation in idiopathic pulmonary arterial hypertension, listed in Table 7 as Wang X-X et al.

**Table 7:** Top 10 co-cited references based on the number of citations.

Rank	Co-cited References	Author	Citations	Journal
1	Rescue of monocrotaline-induced pulmonary arterial hypertension using bone marrow-derived endothelial-like progenitor cells	Zhao Yidan D.	151	Circulation Research
2	Transplantation of autologous endothelial progenitor cells may be beneficial in patients with idiopathic pulmonary arterial hypertension	Wang Xing-Xiang	122	Journal of the American College of Cardiology
3	Isolation of putative progenitor endothelial cells for angiogenesis	Asahara Takayuki	86	Science
4	Evidence of dysfunction of endothelial progenitors in pulmonary arterial hypertension	Toshner Mark	68	American Journal of Respiratory and Critical Care Medicine
5	Safety and efficacy of autologous endothelial progenitor cells transplantation in children with idiopathic pulmonary arterial hypertension: open-label pilot study	Zhu Jun Hui	68	Pediatric Transplantation
6	Exosomes mediate the cytoprotective action of mesenchymal stromal cells on hypoxia-induced pulmonary hypertension	Lee Changjin	67	Circulation
7	Updated clinical classification of pulmonary hypertension	Simonneau Gerald	65	Journal of the American College of Cardiology
8	Reversal of experimental pulmonary hypertension by PDGF inhibition	Schermuly R. T.	64	Journal of Clinical Investigation
9	Exuberant endothelial cell growth and elements of inflammation are present in plexiform lesions of pulmonary hypertension	Tuder R. M.	64	American Journal of Pathology
10	Heterozygous germline mutations in BMPR2, encoding a TGF-β receptor, cause familial primary pulmonary hypertension	Lane Kirk B.	63	Nature Genetics

CiteSpace cluster analysis identified 11 principal clusters (Figure 7B). Cluster #0 (new therapeutic paradigm) was the largest, followed by #1 (systemic sclerosis), #2 (endothelial progenitor cells), and #6 (extracellular vesicles). The timeline visualization showed a progression from early-stage cytological characteristics (#2) through clinicopathological investigations (#1) toward subcellular therapeutic approaches (#6). Clusters #6 (extracellular vesicles) and #7 (gut microbiota) carried warm-toned nodes, indicating recent activity.

Citation burst analysis (Figure 7C) revealed Zhao YD (2005) with the highest burst intensity (26.76). After 2015, guideline documents by Galiè N (2016) and Simonneau G (2019) exhibited high burst intensities, indicating integration of stem cell research into mainstream clinical frameworks. Humbert M (2019), a further consensus document on the diagnosis and classification of pulmonary hypertension, also remained in active burst through 2024.

### 3.7 Keyword co-occurrence, clustering, and burst analysis

Keyword co-occurrence analysis (Figure 8A, Table 8) identified “pulmonary arterial hypertension” as the dominant keyword (434 occurrences, TLS = 1,959). Other high-frequency keywords included “endothelial progenitor cells” (176), “progenitor cells” (142), “expression” (133), “mesenchymal stromal cells” (123), “smooth muscle cells” (118), and “inflammation” (97). Temporal color mapping (Figure 8A) showed a shift from blue-purple nodes (early phase: “gene therapy,” “transplantation,” “bone marrow”) toward orange-red nodes (recent phase: “extracellular vesicles,” “exosomes,” “endothelial-mesenchymal transition”).

**Figure 8:**
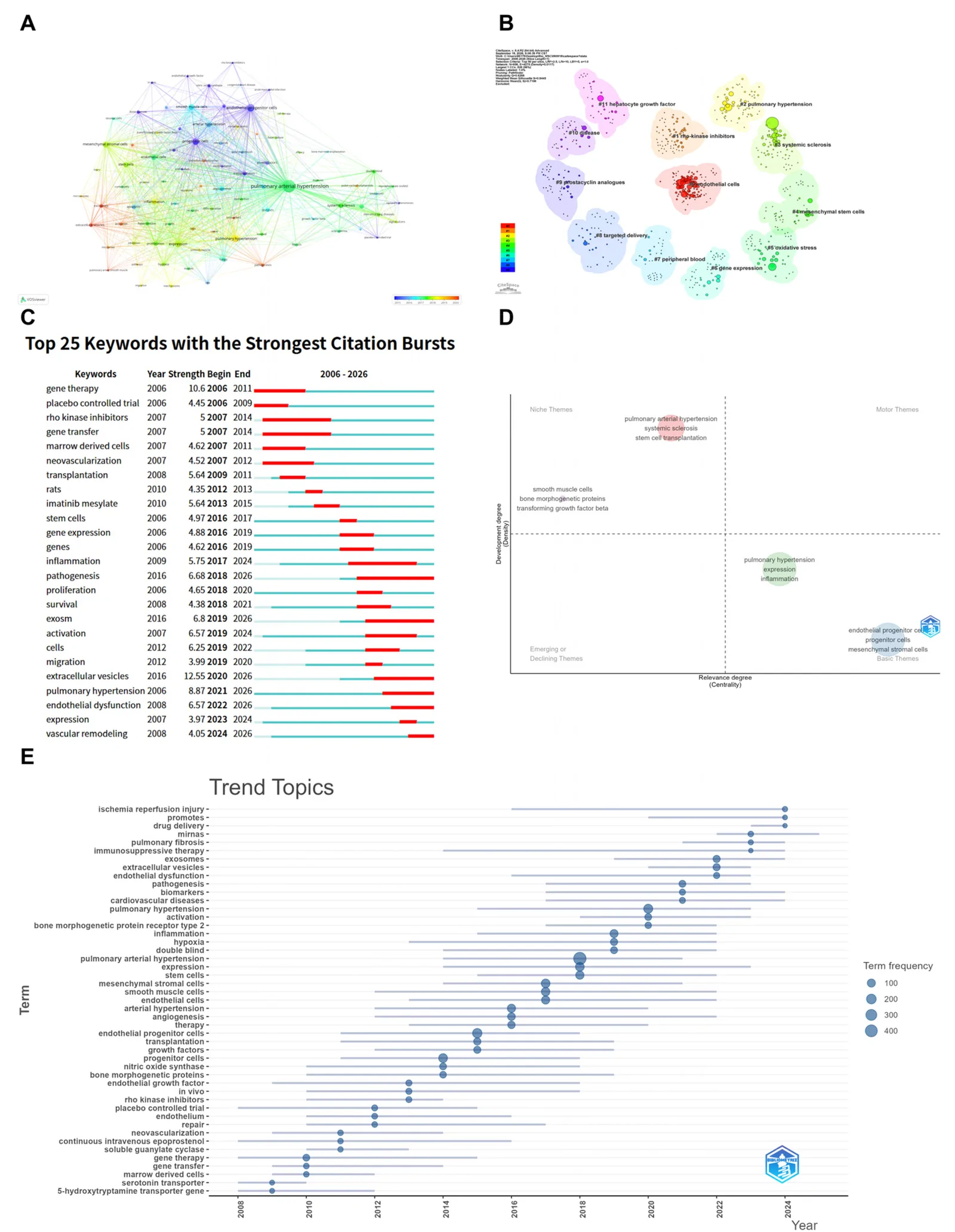
Keyword analysis and research hotspot evolution in stem cell therapy for PAH. **(A)** Keyword co-occurrence network, colored by mean publication year (blue: earlier; red: recent). **(B)** Keyword cluster map (*Q* = 0.6268, *S* = 0.8445). Clusters are labelled by log-likelihood ratio. Cluster labels are reproduced exactly as generated by CiteSpace; the label on cluster #4, “mesenchymal stem cells”, denotes the cell population referred to as mesenchymal stromal cells throughout the text and in Tables 7, 8, and the burst label “exosm” denotes exosomes, the term used in the text. **(C)** Top 25 keywords with the strongest citation bursts (2006–2026). **(D)** Thematic map categorizing research topics by centrality and density. The niche quadrant (upper left) holds pulmonary arterial hypertension, systemic sclerosis and stem cell transplantation, together with smooth muscle cells, bone morphogenetic proteins and transforming growth factor beta. The basic quadrant (lower right) holds pulmonary hypertension, expression and inflammation, together with endothelial progenitor cells, progenitor cells and mesenchymal stromal cells. No theme falls in the motor or the emerging-or-declining quadrant. **(E)** Trend topics chart displaying keyword activity periods and frequency. Early terms such as gene therapy, gene transfer and serotonin transporter were active around 2008–2012, while extracellular vesicles, exosomes and endothelial mesenchymal transition remain active through 2024, consistent with their status as the most rapidly growing research frontiers.

**Table 8:** Top 20 frequency keywords.

Rank	Keyword	Occurrences	Total link strength	Rank	Keyword	Occurrences	Total link strength
1	Pulmonary arterial hypertension	434	1,959	11	Endothelial cells	95	528
2	Endothelial progenitor cells	176	842	12	Systemic sclerosis	85	476
3	Pulmonary hypertension	153	722	13	Proliferation	80	480
4	Progenitor cells	142	726	14	Angiogenesis	79	474
5	Expression	133	738	15	Stem cell transplantation	76	375
6	Mesenchymal stromal cells	123	667	16	Lungs	71	442
7	Smooth muscle cells	118	608	17	Therapy	66	348
8	Arterial hypertension	107	543	18	Apoptosis	64	378
9	Stem cells	107	524	19	Transplantation	62	370
10	Inflammation	97	511	20	Hypoxia	59	345

CiteSpace keyword cluster analysis using the LLR algorithm identified 12 labelled clusters within the 826-node main component, alongside eight smaller ones (Figure 8B). Cluster #0 (endothelial cells) was the largest, holding 112 nodes at the centre of the map. The disease and clinical themes are carried by #2 (pulmonary hypertension, 63 nodes), #3 (systemic sclerosis, 63) and #10 (disease, 38); the therapeutic and delivery themes by #1 (rho-kinase inhibitors, 74), #4 (mesenchymal stromal cells, 54), #8 (targeted delivery, 41) and #9 (prostacyclin analogues, 40); and the mechanistic themes by #5 (oxidative stress, 53), #6 (gene expression, 50) and #11 (hepatocyte growth factor, 37). Cluster #7 (peripheral blood, 41) reflects the circulating-cell and biomarker perspective. The mean publication year of the articles in each cluster separates the classical pharmacology from the cell-based themes: the earliest are prostacyclin analogues (2012.5) and rho-kinase inhibitors (2013.1), while endothelial cells, pulmonary hypertension and systemic sclerosis are the most recent at 2017.3, together with mesenchymal stromal cells (2017.7) and gene expression (2017.9); this ordering matches the year colouring of the figure.

Keyword burst analysis (Figure 8C) revealed three temporal phases: (i) 2006–2011: “gene therapy” (burst intensity 10.60), “transplantation,” and “marrow derived cells” dominated; (ii) 2012–2019: “survival,” “activation,” and “inflammation” emerged; (iii) 2020–2026: “extracellular vesicles” exhibited the highest overall burst intensity (12.55), followed by “pulmonary hypertension” (8.87, 2021–2026), with “endothelial dysfunction” (6.57) and “vascular remodeling” (4.05) remaining in active burst status through 2026.

Strategic diagram analysis (Figure 8D) separated the clinical themes into the niche quadrant and the cell and mechanism terms into the basic quadrant, with no theme in the motor or the emerging-or-declining quadrants. Thematic evolution analysis (Figure 8E) documented the longitudinal migration from “gene therapy” (pre-2010) through “inflammation” and “angiogenesis” (2014–2018) to “extracellular vesicles,” “exosomes,” “microrna,” and “endothelial mesenchymal transition” (post-2019).

## 4 Discussion

### 4.1 Main findings and research evolution

This subsection addresses RQ1 and RQ2. The present analysis shows that research on stem cell-based and cell-free therapies for pulmonary arterial hypertension (PAH) has followed a logistic growth trajectory (*R*^2^ = 0.860) with a theoretical inflection point around 2020.1. With cumulative output reaching 891 records and annual output flat since 2013, annual output has held its level since 2013. The fitted saturation capacity was 1,391 publications (95% CI 1,192–1,687), which varies by 129% across truncation points. During the early exploratory phase (2006–2012), this growth coincided with foundational advances in endothelial progenitor cell biology (24), and the marked increase in citation activity observed around 2017 likely reflects the emergence of several influential studies that contributed to the increasing recognition of stem cell-based approaches as a potential therapeutic strategy in PAH. More importantly, this trajectory was not purely quantitative: co-citation, citation burst, keyword co-occurrence, and thematic evolution analyses support a descriptive three-phase framework of the field's development—from EPC-based cellular replacement (2006–2012) through the expansion of MSC research (2013–2021) to EV/exosome-based cell-free strategies (2022–2026), a phase dated from the final boundary used here although the extracellular vesicle burst begins in 2020 (Figure 8C). This framework, spanning cell-based implantation, cellular secretion products, and nanoparticle-scale EV therapeutics, constitutes the central finding of the present study and distinguishes it from descriptive bibliometric summaries; prior PAH bibliometric analyses did not treat this stem-cell-to-cell-free arc as a dedicated, continuous research front (18). The composition analysis makes the migration a measured change in the distribution of research effort rather than an impression from term salience: over the same period the share of records naming endothelial progenitor cells fell by two thirds while the share naming extracellular vesicles or exosomes rose more than twentyfold. The stabilization of annual output after 2022 may partly reflect normalization after the COVID-19-related surge of 2020–2021 documented across the broader scientific literature (25), and may signal a shift from exploratory investigations toward clinically oriented, mechanism-focused research, although in the absence of independent indicators these interpretations remain speculative and require confirmation through complementary data sources (18, 26, 27).

The collaborative landscape was highly concentrated, with the United States (30.64%) and China (25.03%) together contributing over half of the total output and the United States acting as the central hub of the international network. Some countries with higher multiple-country publication ratios also recorded higher average citation counts per publication—the United Kingdom (MCP 43.48%) recorded the highest average citations per publication (103.5) among major contributors, whereas China's low MCP ratio (14.80%) paralleled its comparatively modest citation impact—although whether this association reflects a genuine collaboration benefit or a self-selection effect cannot be established from bibliometric data alone—multinational teams plausibly benefit from complementary expertise, access to larger patient cohorts, and visibility across distinct regional citation networks (28). At the institutional level, a hub-and-spoke structure centered on Stanford University coexisted with lower-output but disproportionately high-impact centers such as the University of Giessen (2,432 citations from 13 publications), indicating that scientific influence is not determined solely by productivity. Confounding factors such as language barriers, journal preference distributions, and research topic selection may also contribute to the observed country-level patterns and cannot be excluded on the basis of available data.

The field is characterized by stable output since 2013 and expanding international collaboration. By treating the full arc of stem-cell-to-cell-free evolution in PAH as a dedicated analytical target, the present study complements macro-level bibliometric perspectives on cell and gene therapy (29) and provides disease-specific, evidence-grounded orientation for research prioritisation and trial design.

### 4.2 Evolution of stem cell-based therapeutic strategies in PAH

This subsection addresses RQ3. Co-citation networks, citation burst analyses, and keyword evolution collectively support a descriptive three-phase framework (Table 9): EPC-mediated cellular replacement, the expansion of MSC research, and EV/exosome-based cell-free precision therapy. The intellectual foundations of this evolution are reflected in the co-cited author network, where clinical standards (Humbert M., Galiè N.) and basic pathophysiological research (Tuder R.M., Rabinovitch M., Stenmark K.R.) form densely interconnected clusters (1), and in the journal dual-map overlay, whose two dominant paths both terminate in Molecular, Biology, Genetics, one from molecular biology and immunology and one from medicine and clinical medicine, so the clinical as well as the molecular citing literature draws on molecular and genetic knowledge.

**Table 9:** Three-phase framework of stem cell-based and cell-free therapies for PAH (2006–2026).

Development phase	Core therapeutic vehicle	Bibliometric signature	Dominant biological concept	Major translational limitation
2006–2012 (Nascent Exploration)	EPCs with emerging MSC studies	Zhao YD (2005) and Wang XX (2007) as central co-citation nodes; Zhao YD burst intensity 26.76 (2007–2010), highest across all references; Wang XX burst 24.59 (2008–2012). Stage output 179 papers	Cellular replacement and vascular repair	Poor engraftment and low cell retention
2013–2021 (MSC Expansion)	MSCs	Output holds a level from 2013; 482 papers against 179 in the preceding stage; mesenchymal stromal cell share rises from 10.6% to 18.9%	Paracrine signaling and immunomodulation	Product standardization and long-term safety
2022–2026 (Translational Consolidation)	EVs/Exosomes	Reference cluster #0 (new therapeutic paradigm); keyword burst: extracellular vesicles 12.55 (2020–2026), highest keyword burst overall; exosomes 6.80 (2019–2026). Stage output 230 papers	Cell-free precision regulation	Manufacturing scalability, targeting efficiency, and limited large-scale clinical evidence

Only the first boundary is detected as a change point; the phases are successive rather than discrete.

Stage I: EPC-based cellular replacement (2006–2012). Early research was grounded in the concept that transplanted endothelial progenitor cells could directly repair the injured pulmonary endothelium through engraftment and angiogenesis (30). This cellular-replacement rationale dominated the field's formative years, as reflected bibliometrically by the foundational work of Asahara et al. (24), the central co-citation positions of Zhao et al. (8) and Wang et al. (30), and the highest citation burst intensity in the dataset (Zhao Y.D., 26.76). Subsequent mechanistic studies showed that EPC-derived conditioned medium attenuated pulmonary microvascular endothelial cell apoptosis, pointing to a paracrine component of EPC-mediated cytoprotection (31); however, poor long-term engraftment, low pulmonary retention, and concerns regarding unregulated differentiation and tumorigenicity restricted the translational potential of these approaches (10, 11, 32). To date, clinical evaluation of EPC-based therapy for PAH has been limited, and no phase III randomized trial has been completed for any stem cell- or EV-based product in this indication (32, 33).

Stage II: the expansion of MSC research (2013–2021). A major conceptual shift emerged in the mid-2010s when accumulating evidence demonstrated that the therapeutic benefits of stem cells were mediated predominantly through paracrine signaling rather than direct cellular replacement (10, 11, 34). MSC research expanded rapidly in this period, reflecting the immunomodulatory potency, anti-inflammatory and anti-remodeling properties and favorable clinical safety profile of these cells (10, 11). The mechanistic basis of MSC-mediated pulmonary vascular protection is now understood to be multimodal, and can be summarized by mechanism class rather than individual molecules: paracrine and trophic factor secretion sustains endothelial survival and angiogenic regulation; immunomodulation, including polarization of pulmonary macrophages toward an anti-inflammatory phenotype, dampens perivascular inflammation; MSC-derived exosomes suppress pulmonary artery smooth muscle cell proliferation through pathway-specific regulation—including inhibition of EGFR/ErbB2 heterodimerization and Hsp90aa1/ERK signaling, and restoration of BMPR2 signaling with suppression of its endogenous antagonist (35–37); and functional mitochondria can be transferred to stressed pulmonary endothelial cells by mesenchymal stromal cells, restoring bioenergetic capacity (16, 17), and delivery of isolated mitochondria is likewise protective in experimental hypoxic pulmonary hypertension (38). This transition, which the keyword burst analysis captures through the emergence of “survival”, “activation”, and “inflammation” as dominant terms during 2012–2019, and which is attested by the high co-citation ranking of the seminal exosome study of Lee et al. (34), marked a fundamental change in scientific thinking: attention shifted from the transplanted cells themselves toward the bioactive factors they secrete. These pathway-specific findings transformed the therapeutic rationale: attention shifted from stochastic cellular engraftment toward identifying and harnessing the specific molecular effectors—particularly microRNAs and proteins—that mediate the anti-remodeling activity of the MSC secretome (35–37). The identification of a six-microRNA signature in MSC-conditioned medium that modulates endothelial function and provides mechanistic insight into EV-mediated pulmonary endothelial repair (9) further illustrates this movement toward molecularly defined secretome effectors.

Stage III: EV/exosome-based cell-free strategies (2022–2026). This phase is dated from the final boundary used here, and the underlying signal precedes it: the burst for extracellular vesicles begins in 2020 and runs to 2026 (Figure 8C). The most recent stage is characterized by the rapid rise of extracellular vesicles and exosome-based therapies. The largest keyword cluster is #0, endothelial cells, consistent with the endothelial focus of this literature, while extracellular vesicles exhibited the strongest keyword burst in the dataset (12.55) and remained among the most active terms in the trend topics after 2019; exosomes showed a similarly robust burst signal (6.80). EVs recapitulate many of the anti-inflammatory, anti-proliferative, and pro-angiogenic effects of their parent cells (12, 13, 34), and exosome-mediated cytoprotection has been demonstrated in experimental pulmonary hypertension (34). Moreover, the persistent translational bottlenecks of living-cell products—including the absence of standardized characterization criteria, inter-donor variability in MSC potency, and the regulatory complexity of classifying living cell products—have coincided with the pivot toward cell-free strategies (10, 33). As non-living biologic products, EVs can be characterized and reported according to the standardized framework of the updated MISEV2023 guidelines, an important step toward their credible development (39). These developments suggest that the field is systematically transitioning from empirical cell transplantation toward mechanism-driven and precision-engineered regenerative interventions.

The evolution from EPCs to MSCs and subsequently to EVs is consistent with a broader transformation in regenerative medicine, although bibliometric evidence alone cannot distinguish whether this trajectory reflects genuine biological discovery, shifting academic trends, or evolving funding priorities. Contemporary research increasingly emphasizes molecular mechanisms, targeted regulation, and rational bioengineering design. It should also be noted that the phases are not strictly discrete: EPC-related publications continue to appear throughout the study period, and the mesenchymal stromal cell share, having peaked in the middle period, remains substantial in the most recent one.

### 4.3 Emerging frontiers and translational implications

This subsection addresses RQ4 and RQ5. Keyword burst detection, cluster analysis, timeline visualization, and strategic coordinate mapping indicate that the research frontier is moving from proof-of-concept studies toward precision-oriented, mechanism-driven investigations. We restrict this interpretation to directions directly supported by our quantitative indicators.

Extracellular vesicles and exosomes represent the most prominent frontier in the bibliometric record. Their bibliometric dominance in the post-2019 landscape is paralleled by a rapidly diversifying engineering toolbox: recent studies have moved beyond native MSC-derived EVs toward engineered platforms incorporating surface functionalization, pharmacological preconditioning, and genetic cargo modulation for targeted pulmonary delivery (40–44). The second frontier that these data place in the foreground is endothelial-to-mesenchymal transition (EndMT), which has a clear presence in the post-2019 thematic evolution map. First established in PAH by Ranchoux et al. (45), EndMT is now recognized as a key driver of pulmonary vascular remodeling. The increasing prominence of EndMT-related terms reflects growing recognition that pulmonary vascular remodeling is not solely a consequence of abnormal cellular proliferation but also involves dynamic phenotypic reprogramming of endothelial cells, and its regulation by TGF-β/Smad2/3 signaling and BMPR2 deficiency (46), as well as by shear stress-induced ERG loss (47), has been progressively clarified, with transcription factor SOX17 emerging as an endogenous gatekeeper of endothelial identity (46) and the extracellular matrix protein TGFBI promoting EndMT through feed-forward TGF-β signaling, suggesting utility as both a biomarker and a therapeutic target (48). Endothelial dysfunction (burst 6.57) and vascular remodeling (burst 4.05), both remaining in active burst status through 2026, together with microRNA-mediated regulation emerging in the post-2019 thematic map, complete the set of research fronts these data place in the foreground. The convergence of these five themes—EVs and exosomes, EndMT, endothelial dysfunction, vascular remodeling, and microRNA-mediated regulation—suggests an emerging research architecture in which EV-based delivery platforms, endothelial phenotypic regulation, and RNA-mediated mechanisms collectively characterize the recent frontier captured by these analyses, and indicate where regenerative medicine effort is concentrating.

### 4.4 Clinical translation challenges and future directions

Despite two decades of preclinical progress—largely accumulated in monocrotaline and Sugen/hypoxia models (12, 13, 34)—clinical translation remains at an early stage: EPC-based trials registered in PAH remain limited in number, and no phase III randomized trial has been completed for any stem cell- or EV-based product in this indication (32, 33). From a cell and gene therapy development perspective, the PAH field exemplifies the translational trajectory from first-generation cell replacement toward engineered biologic products. Several interlocking challenges must be resolved. First, manufacturing standardization: achieving scalable, reproducible production of MSC-based products remains challenging (10, 33), and compliance with MISEV2023 reporting standards will be essential for the credible development of EV-based products (39). Second, dosing, delivery route, and targeting efficiency: achieving cell-type-specific pulmonary delivery of therapeutic cargo—particularly to diseased endothelial and smooth muscle cells—remains a key engineering objective, toward which surface-functionalized and cargo-engineered EV platforms are among the actively investigated approaches (40–44). Third, long-term safety and efficacy, particularly with regard to pulmonary vascular remodeling and right ventricular function, require evaluation in adequately powered clinical studies (10, 11, 33). Fourth, regulatory frameworks will need to accommodate the unique features of EV-based therapeutics, which differ from both small molecules and living cell products. Looking further ahead, cell-type-resolved single-cell atlases of the diseased pulmonary microenvironment—a literature-informed outlook whose bibliometric signature remains nascent—could provide the molecular map against which the targeting specificity of engineered EVs is rationally designed (49–51). The bibliometric record may help identify the areas attracting the most rapidly increasing research attention: EV engineering, EndMT-targeted interventions, and RNA-mediated regulation, which these data place at the field's most dynamic boundaries.

## 5 Limitations

This study has several limitations. First, bibliometric databases capture a lagged and incomplete representation of scientific activity: preprints, conference proceedings, and industry-sponsored research are underrepresented, and the 2026 data reflect only publications indexed through March 15. Second, our logistic growth model (*R*^2^ = 0.860) provides a useful approximation but may not fully account for external perturbations that may have transiently affected publication rates, nor for potential paradigm-disrupting discoveries capable of resetting the carrying capacity. The fitted saturation capacity is in any case unstable, moving between 607 and 1,391 across truncation points, and is reported as indicative only. Third, the WoSCC-priority integration strategy, while preserving the cited-reference integrity essential for co-citation analysis, resulted in the exclusion of 7 PubMed records (4.7%) that could not be matched via DOI, introducing a minor selection bias. The relevance screen was applied to the 155 records carrying no PAH or PH term; records carrying one were not screened further. Fourth, geographic analyses are constrained by database coverage biases, particularly for non-English-language publications and contributions from low-resource countries. Fifth, co-citation clusters and keyword bursts reflect historical accumulation and may not perfectly anticipate the near-term future. Sixth, our analysis did not distinguish between original articles and reviews, which may exhibit different citation patterns. Seventh, bibliometric trajectories reflect publication activity rather than mechanistic disease progression; the paradigm shifts documented here describe shifts in research emphasis as captured by publication records, not necessarily corresponding shifts in biological understanding or clinical practice. Eighth, we acknowledge that citation counts reflect visibility and consensus rather than intrinsic methodological quality, and may be influenced by self-citation, review articles, and controversy-driven attention. Ninth, although the corpus is PAH-centred rather than PAH-restricted, it necessarily retains studies of broader cell-therapy contexts that cite this literature, and such studies surface among the smaller keyword clusters and may introduce thematic noise there. These caveats notwithstanding, the convergence of multiple complementary bibliometric indicators toward a consistent three-phase narrative supports the descriptive framework used here.

## 6 Conclusion

In conclusion, two decades of research have carried the PAH-stem cell field from cell-based implantation strategies to cell-free precision therapeutics along a bibliometrically traceable trajectory—a pattern that reflects publication activity and research emphasis rather than an inherent directionality in disease understanding. The sustained level of annual output since 2013, the restructuring of collaboration networks, and the reorientation of the thematic lexicon collectively indicate that this is not merely a growing field but one undergoing thematic change. The descriptive EPC → MSC → EV framework documented here provides a bibliometric account of how research emphasis has shifted—particularly EV engineering, EndMT-targeted interventions, and the single-cell-resolution mapping of intercellular communication—which the bibliometric record places at the field's most dynamic boundary. The EV-exosome frontier, the strongest keyword burst in the dataset and the most persistent signal in the recent trend topics, has emerged as a major recent research focus and, we anticipate, a platform for the next wave of translational investigation, with endothelial-to-mesenchymal transition as its most active mechanistic companion.

## Data Availability

The raw data supporting the conclusions of this article will be made available by the authors, without undue reservation.

## Supplementary material

Supplementary Table S1 Complete search strings as executed in the Web of Science Core Collection and PubMed, with the retrieval date, together with the retrieval flowchart inputs. The strings are reproduced exactly as run, which is why the phrase “mesenchymal stem cells” appears in them; the standardized term “mesenchymal stromal cells” is used throughout the manuscript.

Supplementary Table S2 Software and settings for each analysis. For every network and chart, the software and its version, the counting method, the normalization, the node inclusion threshold, the CiteSpace selection and clustering settings where applicable, and the resulting network size and cluster quality metrics *Q* and *S*. The VOSviewer thresholds are the minima of the exported node tables and the item counts their row counts; the CiteSpace parameters are read from the headers printed on the exported maps.

Supplementary Note S1 Composition of the corpus and the phase boundaries. The therapeutic composition of the corpus by stage, its sensitivity to the tagging rule, and the change-point analysis of annual output and of each share series, referred to from Section 3.1.

Supplementary Figure S1 Therapeutic class composition and change points, 2006–2025. **(A)** Annual share of records naming endothelial progenitor cells, mesenchymal stromal cells, or extracellular vesicles and exosomes. A record is tagged from its own keyword fields and may carry more than one tag, so the three shares do not sum to 100. Shaded bands mark the profile 95% interval on each change point and the dotted line marks its least-squares location. The endothelial progenitor cell share changes at 2015 (interval 2013–2015, Davies *p* = 0.0001) and the extracellular vesicle share at 2014 (interval 2010–2016, *p* = 0.039); the mesenchymal stromal cell series supports no change point (*p* = 0.056) and is drawn without one. Its share rises from 10.6% in the first stage to 18.9% in the second and holds at 17.1% in the third, a level shift the series is too noisy to locate as a change point. **(B)** Annual output, with a change point at 2013 (interval 2011–2015, *p* < 0.0001) and a BIC improvement of 30.5 that survives penalising the breakpoint threefold.
