# Supplementary material for: Stem cells, extracellular vesicles, and cell-free strategies in pulmonary arterial hypertension: a bibliometric study, 2006–2026
Source: Front Med (Lausanne). 2026 Oct 27;13:1965787. doi: 10.3389/fmed.2026.1965787 (PMC13748447; doi:10.3389/fmed.2026.1965787)
Supplement: Supplementary file 1 [file Table1.docx]

**Table S1. Executable search strings for the two databases (search date: 15 March 2026).**

1. Web of Science Core Collection

Field: Topic (TS). Document-type and language limits are embedded in the query.

TS=(

(

"mesenchymal stem cell*" OR

"mesenchymal stromal cell*" OR

"bone marrow stem cell*" OR

"bone marrow-derived cell*" OR

"bone marrow mononuclear cell*" OR

"BMMNC*" OR

"endothelial progenitor cell*" OR

"induced pluripotent stem cell*" OR

"iPSC*" OR

"embryonic stem cell*" OR

"adipose-derived stem cell*" OR

"adipose stem cell*" OR

"umbilical cord stem cell*" OR

"UC-MSC*" OR

"Wharton's jelly stem cell*" OR

"Wharton jelly mesenchymal*" OR

"hematopoietic stem cell*" OR

"CD34+ cell*" OR

"CD133+ cell*" OR

"progenitor cell*" OR

"cell therap*" OR

"cell transplant*" OR

"cell-based therap*" OR

"cell infusion" OR

"cell injection" OR

"extracellular vesicle*" OR

"exosome*" OR

"microvesicle*" OR

"conditioned medium" OR

"conditioned media" OR

"secretome" OR

"paracrine effect*" OR

"cell-free therap*" OR

"eNOS-transfected cell*" OR

"gene-modified stem cell*"

)

AND

(

"pulmonary arterial hypertension" OR

"idiopathic pulmonary arterial hypertension" OR

"IPAH" OR

"heritable pulmonary arterial hypertension" OR

"HPAH" OR

"familial pulmonary arterial hypertension" OR

"FPAH" OR

"drug-induced pulmonary arterial hypertension" OR

"PAH associated with connective tissue disease" OR

"PAH associated with congenital heart disease" OR

"PAH associated with HIV" OR

"precapillary pulmonary hypertension" OR

"WHO group 1 pulmonary hypertension" OR

"group 1 pulmonary hypertension" OR

"mean pulmonary arterial pressure" OR

"mPAP" OR

"pulmonary vascular resistance" OR

"pulmonary arterial remodel*" OR

"pulmonary arteriopathy" OR

"pulmonary artery smooth muscle cell*" OR

"PASMC*" OR

"pulmonary artery endothelial cell*" OR

"PAEC*" OR

"BMPR2" OR

"bone morphogenetic protein receptor type 2" OR

"monocrotaline-induced pulmonary arterial hypertension" OR

"MCT-induced pulmonary arterial hypertension" OR

"Sugen5416 hypoxia" OR

"SuHx pulmonary arterial hypertension" OR

"plexiform lesion*"

)

)

AND DT=(Article OR Review)

AND LA=(English)

2. PubMed (NCBI)

Controlled vocabulary (MeSH) combined with free text restricted to the title and abstract [TIAB].

(

(

"Stem Cells"[MeSH] OR

"Stem Cell Transplantation"[MeSH] OR

"Mesenchymal Stem Cells"[MeSH] OR

"Hematopoietic Stem Cells"[MeSH] OR

"Embryonic Stem Cells"[MeSH] OR

"Induced Pluripotent Stem Cells"[MeSH] OR

"Endothelial Progenitor Cells"[MeSH] OR

"Extracellular Vesicles"[MeSH] OR

"Exosomes"[MeSH] OR

"stem cell*"[TIAB] OR

"stem cell therapy"[TIAB] OR

"stem cell transplantation"[TIAB] OR

"mesenchymal stem cell*"[TIAB] OR

"mesenchymal stromal cell*"[TIAB] OR

"bone marrow stem cell*"[TIAB] OR

"bone marrow-derived cell*"[TIAB] OR

"bone marrow mononuclear cell*"[TIAB] OR

"BMMNC"[TIAB] OR

"endothelial progenitor cell*"[TIAB] OR

"induced pluripotent stem cell*"[TIAB] OR

"iPSC*"[TIAB] OR

"embryonic stem cell*"[TIAB] OR

"adipose-derived stem cell*"[TIAB] OR

"adipose stem cell*"[TIAB] OR

"umbilical cord stem cell*"[TIAB] OR

"UC-MSC"[TIAB] OR

"Wharton's jelly stem cell*"[TIAB] OR

"Wharton jelly mesenchymal*"[TIAB] OR

"hematopoietic stem cell*"[TIAB] OR

"CD34+ cell*"[TIAB] OR

"CD133+ cell*"[TIAB] OR

"progenitor cell*"[TIAB] OR

"cell therap*"[TIAB] OR

"cell transplant*"[TIAB] OR

"cell-based therap*"[TIAB] OR

"cell infusion"[TIAB] OR

"extracellular vesicle*"[TIAB] OR

"exosome*"[TIAB] OR

"microvesicle*"[TIAB] OR

"conditioned medium"[TIAB] OR

"conditioned media"[TIAB] OR

"secretome"[TIAB] OR

"paracrine effect*"[TIAB] OR

"cell-free therap*"[TIAB] OR

"eNOS-transfected"[TIAB] OR

"gene-modified stem cell*"[TIAB]

)

AND

(

"Familial Primary Pulmonary Hypertension"[MeSH] OR

"Pulmonary Arterial Hypertension"[MeSH] OR

"pulmonary arterial hypertension"[TIAB] OR

"idiopathic pulmonary arterial hypertension"[TIAB] OR

"IPAH"[TIAB] OR

"heritable pulmonary arterial hypertension"[TIAB] OR

"HPAH"[TIAB] OR

"familial pulmonary arterial hypertension"[TIAB] OR

"FPAH"[TIAB] OR

"drug-induced pulmonary arterial hypertension"[TIAB] OR

"connective tissue disease-associated pulmonary arterial hypertension"[TIAB] OR

"systemic sclerosis-associated pulmonary arterial hypertension"[TIAB] OR

"scleroderma-associated pulmonary arterial hypertension"[TIAB] OR

"congenital heart disease-associated pulmonary arterial hypertension"[TIAB] OR

"Eisenmenger syndrome"[TIAB] OR

"HIV-associated pulmonary arterial hypertension"[TIAB] OR

"WHO group 1 pulmonary hypertension"[TIAB] OR

"group 1 pulmonary hypertension"[TIAB] OR

"pulmonary arterial remodel*"[TIAB] OR

"pulmonary arteriopathy"[TIAB] OR

"pulmonary artery smooth muscle cell*"[TIAB] OR

"PASMC"[TIAB] OR

"pulmonary artery endothelial cell*"[TIAB] OR

"PAEC"[TIAB] OR

"BMPR2"[TIAB] OR

"bone morphogenetic protein receptor type 2"[TIAB] OR

"monocrotaline-induced pulmonary arterial hypertension"[TIAB] OR

"MCT-induced pulmonary arterial hypertension"[TIAB] OR

"Sugen5416 hypoxia"[TIAB] OR

"SuHx"[TIAB] OR

"Sugen hypoxia"[TIAB] OR

"plexiform lesion*"[TIAB]

)

)

AND ("journal article"[PT] OR "review"[PT])

AND (english[lang])

3. Differences between the two disease concepts

Present in WoSCC Concept Group B but not in PubMed:

• mean pulmonary arterial pressure

• mpap

• pah associated with congenital heart disease

• pah associated with connective tissue disease

• pah associated with hiv

• precapillary pulmonary hypertension

• pulmonary vascular resistance

• suhx pulmonary arterial hypertension

Present in PubMed Concept Group B but not in WoSCC:

• congenital heart disease-associated pulmonary arterial hypertension

• connective tissue disease-associated pulmonary arterial hypertension

• eisenmenger syndrome

• familial primary pulmonary hypertension

• hiv-associated pulmonary arterial hypertension

• scleroderma-associated pulmonary arterial hypertension

• sugen hypoxia

• suhx

• systemic sclerosis-associated pulmonary arterial hypertension

Table S1. Complete search strategies used in the Web of Science Core Collection (WoSCC) and PubMed databases.

| **Database** | **Search component** | **Search terms** |
| --- | --- | --- |
| WoSCC | Concept Group A: Stem cells, extracellular vesicles, and cell-free therapies | "mesenchymal stem cell*" OR "mesenchymal stromal cell*" OR "bone marrow stem cell*" OR "bone marrow-derived cell*" OR "bone marrow mononuclear cell*" OR "BMMNC*" OR "endothelial progenitor cell*" OR "induced pluripotent stem cell*" OR "iPSC*" OR "embryonic stem cell*" OR "adipose-derived stem cell*" OR "adipose stem cell*" OR "umbilical cord stem cell*" OR "UC-MSC*" OR "Wharton's jelly stem cell*" OR "Wharton jelly mesenchymal*" OR "hematopoietic stem cell*" OR "CD34+ cell*" OR "CD133+ cell*" OR "progenitor cell*" OR "cell therap*" OR "cell transplant*" OR "cell-based therap*" OR "cell infusion" OR "cell injection" OR "extracellular vesicle*" OR "exosome*" OR "microvesicle*" OR "conditioned medium" OR "conditioned media" OR "secretome" OR "paracrine effect*" OR "cell-free therap*" OR "eNOS-transfected cell*" OR "gene-modified stem cell*" |
| WoSCC | Concept Group B: Pulmonary arterial hypertension | "pulmonary arterial hypertension" OR "idiopathic pulmonary arterial hypertension" OR "IPAH" OR "heritable pulmonary arterial hypertension" OR "HPAH" OR "familial pulmonary arterial hypertension" OR "FPAH" OR "drug-induced pulmonary arterial hypertension" OR "PAH associated with connective tissue disease" OR "PAH associated with congenital heart disease" OR "PAH associated with HIV" OR "precapillary pulmonary hypertension" OR "WHO group 1 pulmonary hypertension" OR "group 1 pulmonary hypertension" OR "mean pulmonary arterial pressure" OR "mPAP" OR "pulmonary vascular resistance" OR "pulmonary arterial remodel*" OR "pulmonary arteriopathy" OR "pulmonary artery smooth muscle cell*" OR "PASMC*" OR "pulmonary artery endothelial cell*" OR "PAEC*" OR "BMPR2" OR "bone morphogenetic protein receptor type 2" OR "monocrotaline-induced pulmonary arterial hypertension" OR "MCT-induced pulmonary arterial hypertension" OR "Sugen5416 hypoxia" OR "SuHx pulmonary arterial hypertension" OR "plexiform lesion*" |
| WoSCC | Final search query | TS=((Concept Group A) AND (Concept Group B)) AND DT=(Article OR Review) AND LA=(English) |
| PubMed | Concept Group A: Stem cells, extracellular vesicles, and cell-free therapies | "Stem Cells"[MeSH] OR "Stem Cell Transplantation"[MeSH] OR "Mesenchymal Stem Cells"[MeSH] OR "Hematopoietic Stem Cells"[MeSH] OR "Embryonic Stem Cells"[MeSH] OR "Induced Pluripotent Stem Cells"[MeSH] OR "Endothelial Progenitor Cells"[MeSH] OR "Extracellular Vesicles"[MeSH] OR "Exosomes"[MeSH] OR "stem cell*"[TIAB] OR "stem cell therapy"[TIAB] OR "stem cell transplantation"[TIAB] OR "mesenchymal stem cell*"[TIAB] OR "mesenchymal stromal cell*"[TIAB] OR "bone marrow stem cell*"[TIAB] OR "bone marrow-derived cell*"[TIAB] OR "bone marrow mononuclear cell*"[TIAB] OR "BMMNC"[TIAB] OR "endothelial progenitor cell*"[TIAB] OR "induced pluripotent stem cell*"[TIAB] OR "iPSC*"[TIAB] OR "embryonic stem cell*"[TIAB] OR "adipose-derived stem cell*"[TIAB] OR "adipose stem cell*"[TIAB] OR "umbilical cord stem cell*"[TIAB] OR "UC-MSC"[TIAB] OR "Wharton's jelly stem cell*"[TIAB] OR "Wharton jelly mesenchymal*"[TIAB] OR "hematopoietic stem cell*"[TIAB] OR "CD34+ cell*"[TIAB] OR "CD133+ cell*"[TIAB] OR "progenitor cell*"[TIAB] OR "cell therap*"[TIAB] OR "cell transplant*"[TIAB] OR "cell-based therap*"[TIAB] OR "cell infusion"[TIAB] OR "extracellular vesicle*"[TIAB] OR "exosome*"[TIAB] OR "microvesicle*"[TIAB] OR "conditioned medium"[TIAB] OR "conditioned media"[TIAB] OR "secretome"[TIAB] OR "paracrine effect*"[TIAB] OR "cell-free therap*"[TIAB] OR "eNOS-transfected"[TIAB] OR "gene-modified stem cell*"[TIAB] |
| PubMed | Concept Group B: Pulmonary arterial hypertension | "Familial Primary Pulmonary Hypertension"[MeSH] OR "Pulmonary Arterial Hypertension"[MeSH] OR "pulmonary arterial hypertension"[TIAB] OR "idiopathic pulmonary arterial hypertension"[TIAB] OR "IPAH"[TIAB] OR "heritable pulmonary arterial hypertension"[TIAB] OR "HPAH"[TIAB] OR "familial pulmonary arterial hypertension"[TIAB] OR "FPAH"[TIAB] OR "drug-induced pulmonary arterial hypertension"[TIAB] OR "connective tissue disease-associated pulmonary arterial hypertension"[TIAB] OR "systemic sclerosis-associated pulmonary arterial hypertension"[TIAB] OR "scleroderma-associated pulmonary arterial hypertension"[TIAB] OR "congenital heart disease-associated pulmonary arterial hypertension"[TIAB] OR "Eisenmenger syndrome"[TIAB] OR "HIV-associated pulmonary arterial hypertension"[TIAB] OR "WHO group 1 pulmonary hypertension"[TIAB] OR "group 1 pulmonary hypertension"[TIAB] OR "pulmonary arterial remodel*"[TIAB] OR "pulmonary arteriopathy"[TIAB] OR "pulmonary artery smooth muscle cell*"[TIAB] OR "PASMC"[TIAB] OR "pulmonary artery endothelial cell*"[TIAB] OR "PAEC"[TIAB] OR "BMPR2"[TIAB] OR "bone morphogenetic protein receptor type 2"[TIAB] OR "monocrotaline-induced pulmonary arterial hypertension"[TIAB] OR "MCT-induced pulmonary arterial hypertension"[TIAB] OR "Sugen5416 hypoxia"[TIAB] OR "SuHx"[TIAB] OR "Sugen hypoxia"[TIAB] OR "plexiform lesion*"[TIAB] |
| PubMed | Final search query | ((Concept Group A) AND (Concept Group B)) AND ("journal article"[PT] OR "review"[PT]) AND (English[lang]) |

Retrieval summary (the inputs to Figure 1)

| WoSCC, identified by the topic search | 1,140 |
| --- | --- |
| removed: document type (meeting abstracts, editorials, book chapters, other) | −127 |
| removed: non-English | −8 |
| WoSCC retained | 1,005 |
| PubMed, identified | 701 |
| removed: publication type or language | −551 |
| PubMed, article or review records retained | 150 |
| not locatable in Web of Science (no accession, excluded) | −7 |
| PubMed records carried forward | 143 |
| Pooled before deduplication (1,005 + 143) | 1,148 |
| removed: duplicates present in both pulls | −126 |
| Unique records after deduplication | 1,022 |
| removed: read in full and judged not relevant to the topic | −131 |
| Final corpus | 891 |
