# Supplementary material for: Stem cells, extracellular vesicles, and cell-free strategies in pulmonary arterial hypertension: a bibliometric study, 2006–2026
Source: Front Med (Lausanne). 2026 Oct 27;13:1965787. doi: 10.3389/fmed.2026.1965787 (PMC13748447; doi:10.3389/fmed.2026.1965787)
Supplement: Supplementary file 2 [file Table2.docx]

**Table S2. Software and settings for each analysis.**

| **Analysis (figure)** | **Software (version)** | **Counting** | **Normalization** | **Inclusion threshold** | **Selection and clustering** | **Result** |
| --- | --- | --- | --- | --- | --- | --- |
| Keyword co-occurrence (Fig. 8A) | VOSviewer 1.6.20 | Full counting | Association strength | ≥ 20 occurrences | — | 90 items |
| Keyword clusters (Fig. 8B) | CiteSpace 6.4.R2 | n/a | n/a | Top 50 per slice | Slices 1 yr; LRF 2.5, L/N 10, LBY 5, e 1.0; pathfinder pruning of sliced networks; LLR labels | N 856, E 4,275, Q 0.6268, S 0.8445 |
| Keyword bursts (Fig. 8C) | CiteSpace 6.4.R2 | n/a | n/a | — | CiteSpace burst detection; 25 strongest shown | — |
| Thematic map (Fig. 8D) | biblioshiny | n/a | n/a | — | — | — |
| Trend topics (Fig. 8E) | biblioshiny | n/a | n/a | — | — | — |
| Co-authorship, authors (Fig. 4A) | VOSviewer 1.6.20 | Full counting | Association strength | ≥ 8 documents | — | 21 items |
| Circular author diagram (Fig. 4B) | Scimago Graphica | n/a | n/a | — | Node and link data exported from VOSviewer | — |
| Co-cited authors (Fig. 4C) | VOSviewer 1.6.20 | Full counting | Association strength | ≥ 20 citations | — | 383 items |
| Author production over time (Fig. 4D) | biblioshiny | n/a | n/a | — | — | Top 10 authors |
| Institutional co-authorship (Fig. 5A) | VOSviewer 1.6.20 | Full counting | LinLog | ≥ 5 documents | — | 73 items |
| Institutional citation (Fig. 5B) | VOSviewer 1.6.20 | Full counting | Association strength | ≥ 5 documents | — | 73 items |
| Country co-authorship network (Fig. 3A) | VOSviewer 1.6.20 | Full counting | Association strength | ≥ 5 documents | — | 24 items |
| Country chord diagram (Fig. 3B) | Charticulator | n/a | n/a | — | Data exported from VOSviewer, converted with Gephi | — |
| Country cluster map (Fig. 3C) | CiteSpace 6.4.R2 | n/a | n/a | Top 50 per slice | Slices 1 yr; LRF 2.5, L/N 10, LBY 5, e 1.0 | N 59, E 262 |
| SCP/MCP bar chart (Fig. 3D) | biblioshiny | n/a | n/a | — | — | Top 20 countries |
| Geographic distribution map (Fig. 3E) | Scimago Graphica | n/a | n/a | — | Node and link data exported from VOSviewer | — |
| Source journals (Fig. 6A) | VOSviewer 1.6.20 | Full counting | Association strength | ≥ 5 documents | — | 40 items |
| Co-cited sources (Fig. 6B) | VOSviewer 1.6.20 | Full counting | Association strength | ≥ 200 citations | — | 58 items |
| Country-source-co-cited source Sankey (Fig. 6C) | biblioshiny | n/a | n/a | — | — | — |
| Journal dual-map overlay (Fig. 6D) | CiteSpace 6.4.R2 | n/a | n/a | — | CiteSpace dual-map overlay | — |
| Co-cited references (Fig. 7A) | VOSviewer 1.6.20 | Full counting | Association strength | ≥ 55 citations | — | 19 items |
| Reference clusters (Fig. 7B) | CiteSpace 6.4.R2 | n/a | n/a | Top 50 per slice | Slices 1 yr; LRF 2.5, L/N 10, LBY 5, e 1.0; no pruning; LLR labels | N 2,474, E 7,755, Q 0.8545, S 0.965 |
| Reference bursts (Fig. 7C) | CiteSpace 6.4.R2 | n/a | n/a | — | CiteSpace burst detection; 25 strongest shown | — |
| Annual output, citations, growth model (Fig. 2) | bibliometrix::lifeCycle, R 4.5.2 | n/a | n/a | Complete calendar years 2006-2025 | Partial 2026 excluded | K 1,391 (95% CI 1,192-1,687); tm 2020.1; R² 0.860 |
| Therapeutic class composition and change points (Fig. S1) | R 4.5.2, segmented 2.2.1 | n/a | n/a | Tags from merged author keywords and Keywords Plus | Poisson model for output, linear for shares; BIC, two- and threefold breakpoint penalties, Davies test, fixed-breakpoint profile | Output break 2013 (2011-2015); EPC 2015 (2013-2015); MSC none; EV 2014 (2010-2016) |
| Term harmonization, all keyword analyses | CiteSpace, VOSviewer and biblioshiny | n/a | n/a | 1,312 rules | Hyphenation, spelling, singular and plural, abbreviations; no semantic merging | Three files, one rule set |

Counting and normalization for the VOSviewer networks were set by the user; the inclusion thresholds are the minima of the exported node tables and the item counts their row counts. CiteSpace parameters are read from the headers of the exported maps, which record the run that produced them. The 2026 partial year is excluded from the growth model and from the change-point analysis.
