# Supplementary material for: Stem cells, extracellular vesicles, and cell-free strategies in pulmonary arterial hypertension: a bibliometric study, 2006–2026
Source: Front Med (Lausanne). 2026 Oct 27;13:1965787. doi: 10.3389/fmed.2026.1965787 (PMC13748447; doi:10.3389/fmed.2026.1965787)
Supplement: Supplementary file 3 [file Table3.docx]

**Supplementary Note S1. Composition of the corpus and the phase boundaries**

This note reports the two analyses referred to from Section 3.1. Both use the same 891-record

corpus as the rest of the manuscript.

**S1.1 Therapeutic composition**

Each record was classified by therapeutic class from its own merged author keywords and

Keywords Plus: endothelial progenitor cell, mesenchymal stromal cell, or extracellular

vesicle and exosome. A compound such as mesenchymal stromal cell derived exosomes is counted

under both classes, so the shares do not sum to 100.

| Stage | n | Endothelial progenitor cell | Mesenchymal stromal cell | Extracellular vesicle |
| --- | --- | --- | --- | --- |
| 2006-2012 | 179 | 33.0% | 10.6% | 1.1% |
| 2013-2021 | 482 | 20.7% | 18.9% | 12.2% |
| 2022-2025 | 217 | 9.2% | 17.1% | 24.9% |

The endothelial progenitor cell share falls across the three stages and the extracellular

vesicle share rises. The mesenchymal stromal cell share rises and then holds: it is 10.6% in

the first stage, 18.9% in the second and 17.1% in the third, and the annual values for

2022-2025, 10.3%, 19.1%, 20.0% and 19.3%, are no lower than those of the middle stage.

The direction of travel is stable under stricter and looser tagging rules: with exact phrase

matching the endothelial progenitor cell share falls 33.0 to 20.7 to 9.2 and the

extracellular vesicle share rises 0.0 to 10.4 to 24.4; with looser rules they fall 60.9 to

34.9 to 18.0 and rise 1.1 to 12.4 to 25.3.

**S1.2 Change points**

Segmented regression (segmented 2.2.1 in R) was applied to annual output and to each share

series, with the number of breakpoints chosen by BIC. Because an estimated breakpoint is not

a fixed regressor and BIC under-penalises one, the comparison was repeated with breakpoints

penalised twofold and threefold, the Davies test was used to ask whether a single straight

line is adequate, and each location was profiled by refitting with the breakpoint fixed.

Annual output has one well-identified change point, at 2013: the Davies test rejects a single

straight line at p < 0.0001, the BIC improvement is 30.5 and survives a threefold penalty, and

refitting with the breakpoint fixed lowers the residual sum of squares by 74 per cent, with a

profile 95 per cent interval of 2011 to 2015. This coincides with the first boundary used in

the text.

The composition series are less sharp. The endothelial progenitor cell share changes at 2015

(interval 2013 to 2015, p = 0.0001). The mesenchymal stromal cell series supports no change

point (p = 0.056, interval 2011 to 2018), and the BIC improvement of 3.0 falls to 0.01 under

a twofold penalty. The extracellular vesicle series rises monotonically with a single

marginal change near 2014 (interval 2010 to 2016, p = 0.039); the two-break model at 2015 and

2021 wins on plain BIC but loses under a twofold penalty, so the second break is not

reported.

The first boundary is therefore detected in the data and the second and third are

descriptive. The extracellular vesicle burst begins in 2020 rather than at the 2022

boundary, as the burst analysis in Section 3.7 reports.
